# Reporting animal research: Explanation and elaboration for the ARRIVE guidelines 2.0

**DOI:** 10.1371/journal.pbio.3000411

**Published:** 2020-07-14

**Authors:** Nathalie Percie du Sert, Amrita Ahluwalia, Sabina Alam, Marc T. Avey, Monya Baker, William J. Browne, Alejandra Clark, Innes C. Cuthill, Ulrich Dirnagl, Michael Emerson, Paul Garner, Stephen T. Holgate, David W. Howells, Viki Hurst, Natasha A. Karp, Stanley E. Lazic, Katie Lidster, Catriona J. MacCallum, Malcolm Macleod, Esther J. Pearl, Ole H. Petersen, Frances Rawle, Penny Reynolds, Kieron Rooney, Emily S. Sena, Shai D. Silberberg, Thomas Steckler, Hanno Würbel

**Affiliations:** 1 NC3Rs, London, United Kingdom; 2 The William Harvey Research Institute, London, United Kingdom; 3 Barts Cardiovascular CTU, Queen Mary University of London, London, United Kingdom; 4 Taylor & Francis Group, London, United Kingdom; 5 Health Science Practice, ICF, Durham, North Carolina, United States of America; 6 Nature, San Francisco, California, United States of America; 7 School of Education, University of Bristol, Bristol, United Kingdom; 8 PLOS ONE, Cambridge, United Kingdom; 9 School of Biological Sciences, University of Bristol, Bristol, United Kingdom; 10 QUEST Center for Transforming Biomedical Research, Berlin Institute of Health & Department of Experimental Neurology, Charite Universitätsmedizin Berlin, Berlin, Germany; 11 National Heart and Lung Institute, Imperial College London, London, United Kingdom; 12 Centre for Evidence Synthesis in Global Health, Clinical Sciences Department, Liverpool School of Tropical Medicine, Liverpool, United Kingdom; 13 Clinical and Experimental Sciences, University of Southampton, Southampton, United Kingdom; 14 Tasmanian School of Medicine, University of Tasmania, Hobart, Australia; 15 Data Sciences & Quantitative Biology, Discovery Sciences, R&D, AstraZeneca, Cambridge, United Kingdom; 16 Prioris.ai Inc, Ottawa, Canada; 17 Hindawi Ltd, London, United Kingdom; 18 Centre for Clinical Brain Sciences, University of Edinburgh, Edinburgh, United Kingdom; 19 Academia Europaea Knowledge Hub, Cardiff University, Cardiff, United Kingdom; 20 Medical Research Council, London, United Kingdom; 21 Statistics in Anesthesiology Research (STAR) Core, Department of Anesthesiology, College of Medicine, University of Florida, Gainesville, Florida, United States of America; 22 Discipline of Exercise and Sport Science, Faculty of Medicine and Health, University of Sydney, Sydney, Australia; 23 National Institute of Neurological Disorders and Stroke, Bethesda, Maryland, United States of America; 24 Janssen Pharmaceutica NV, Beerse, Belgium; 25 Veterinary Public Health Institute, Vetsuisse Faculty, University of Bern, Bern, Switzerland; University Paris Descartes, FRANCE

## Abstract

Improving the reproducibility of biomedical research is a major challenge. Transparent and accurate reporting is vital to this process; it allows readers to assess the reliability of the findings and repeat or build upon the work of other researchers. The ARRIVE guidelines (Animal Research: Reporting In Vivo Experiments) were developed in 2010 to help authors and journals identify the minimum information necessary to report in publications describing in vivo experiments. Despite widespread endorsement by the scientific community, the impact of ARRIVE on the transparency of reporting in animal research publications has been limited. We have revised the ARRIVE guidelines to update them and facilitate their use in practice. The revised guidelines are published alongside this paper. This explanation and elaboration document was developed as part of the revision. It provides further information about each of the 21 items in ARRIVE 2.0, including the rationale and supporting evidence for their inclusion in the guidelines, elaboration of details to report, and examples of good reporting from the published literature. This document also covers advice and best practice in the design and conduct of animal studies to support researchers in improving standards from the start of the experimental design process through to publication.

## Abstract 2

The NC3Rs developed the ARRIVE guidelines in 2010 to help authors and journals identify the minimum information necessary to report in publications describing in vivo experiments. This article explains the rationale behind each item in the revised and updated ARRIVE guidelines 2019, clarifying key concepts and providing illustrative examples.

*See S1 Annotated byline for individual authors’ positions at the time this article was submitted*.

*See S1 Annotated References for further context on the works cited in this article*.

## Introduction

Transparent and accurate reporting is essential to improve the reproducibility of scientific research; it enables others to scrutinise the methodological rigour of the studies, assess how reliable the findings are, and repeat or build upon the work.

However, evidence shows that the majority of publications fail to include key information and there is significant scope to improve the reporting of studies involving animal research [1–4]. To that end, the UK National Centre for the 3Rs (NC3Rs) published the ARRIVE (Animal Research: Reporting In Vivo Experiments) guidelines in 2010. The guidelines are a checklist of information to include in a manuscript to ensure that publications contain enough information to add to the knowledge base [5]. The guidelines have received widespread endorsement from the scientific community and are currently recommended by more than a thousand journals, with further endorsement from research funders, universities, and learned societies worldwide.

Studies measuring the impact of ARRIVE on the quality of reporting have produced mixed results [6–11], and there is evidence that in vivo scientists are not sufficiently aware of the importance of reporting the information covered in the guidelines and fail to appreciate the relevance to their work or their research field [12].

As a new international working group—the authors of this publication—we have revised the guidelines to update them and facilitate their uptake; the ARRIVE guidelines 2.0 are published alongside this paper [13]. We have updated the recommendations in line with current best practice, reorganised the information, and classified the items into two sets. The ARRIVE Essential 10 constitute the minimum reporting requirement, and the Recommended Set provides further context to the study described. Although reporting both sets is best practice, an initial focus on the most critical issues helps authors, journal staff, editors, and reviewers use the guidelines in practice and allows a pragmatic implementation. Once the Essential 10 are consistently reported in manuscripts, items from the Recommended Set can be added to journal requirements over time until all 21 items are routinely reported in all manuscripts. Full methodology for the revision and the allocation of items into sets is described in the accompanying publication [13].

A key aspect of the revision was to develop this explanation and elaboration document to provide background and rationale for each of the 21 items of ARRIVE 2.0. Here, we present additional guidance for each item and subitem, explain the importance of reporting this information in manuscripts that describe animal research, elaborate on what to report, and provide supporting evidence. The guidelines apply to all areas of bioscience research involving living animals. That includes mammalian species as well as model organisms such as *Drosophila* or *Caenorhabditis elegans*. Each item is equally relevant to manuscripts centred around a single animal study and broader-scope manuscripts describing in vivo observations along with other types of experiments. The exact type of detail to report, however, might vary between species and experimental setup; this is acknowledged in the guidance provided for each item.

We recognise that the purpose of the research influences the design of the study. Hypothesis-testing research evaluates specific hypotheses, using rigorous methods to reduce the risk of bias and a statistical analysis plan that has been defined before the study starts. In contrast, exploratory research often investigates many questions simultaneously without adhering to strict standards of rigour; this flexibility is used to develop or test novel methods and generate theories and hypotheses that can be formally tested later. Both study types make valuable contributions to scientific progress. Transparently reporting the purpose of the research and the level of rigour used in the design, execution, and analysis of the study enables readers to decide how to use the research, whether the findings are groundbreaking and need to be confirmed before building on them, or whether they are robust enough to be applied to other research settings.

To contextualise the importance of reporting information described in the Essential 10, this document also covers experimental design concepts and best practices. This has two main purposes: First, it helps authors understand the relevance of this information for readers to assess the reliability of the reported results, thus encouraging thorough reporting. Second, it supports the implementation of best practices in the design and conduct of animal research. Consulting this document at the start of the process when planning an in vivo experiment will enable researchers to make the best use of it, implement the advice on study design, and prepare for the information that will need to be collected during the experiment to report the study in adherence with the guidelines.

To ensure that the recommendations are as clear and useful as possible to the target audience, this document was road tested alongside the revised guidelines with researchers preparing manuscripts describing in vivo research [13]. Each item is written as a self-contained section, enabling authors to refer to particular items independently, and a glossary (Box 1) explains common statistical terms. Each subitem is also illustrated with examples of good reporting from the published literature. Explanations and examples are also available from the ARRIVE guidelines website: https://www.arriveguidelines.org.

**Box 1. Glossary**

**Bias:** The over- or underestimation of the true effect of an intervention. Bias is caused by inadequacies in the design, conduct, or analysis of an experiment, resulting in the introduction of error.

**Descriptive and inferential statistics:** Descriptive statistics are used to summarise the data. They generally include a measure of central tendency (e.g., mean or median) and a measure of spread (e.g., standard deviation or range). Inferential statistics are used to make generalisations about the population from which the samples are drawn. Hypothesis tests such as ANOVA, Mann-Whitney, or *t* tests are examples of inferential statistics.

**Effect size:** Quantitative measure of differences between groups, or strength of relationships between variables.

**Experimental unit:** Biological entity subjected to an intervention independently of all other units, such that it is possible to assign any two experimental units to different treatment groups. Sometimes known as unit of randomisation.

**External validity:** Extent to which the results of a given study enable application or generalisation to other studies, study conditions, animal strains/species, or humans.

**False negative:** Statistically nonsignificant result obtained when the alternative hypothesis (H_1_) is true. In statistics, it is known as the type II error.

**False positive:** Statistically significant result obtained when the null hypothesis (H_0_) is true. In statistics, it is known as the type I error.

**Independent variable:** Variable that either the researcher manipulates (treatment, condition, time) or is a property of the sample (sex) or a technical feature (batch, cage, sample collection) that can potentially affect the outcome measure. Independent variables can be scientifically interesting, or nuisance variables. Also known as predictor variable.

**Internal validity:** Extent to which the results of a given study can be attributed to the effects of the experimental intervention, rather than some other, unknown factor(s) (e.g., inadequacies in the design, conduct, or analysis of the study introducing bias).

**Nuisance variable:** Variables that are not of primary interest but should be considered in the experimental design or the analysis because they may affect the outcome measure and add variability. They become confounders if, in addition, they are correlated with an independent variable of interest, as this introduces bias. Nuisance variables should be considered in the design of the experiment (to prevent them from becoming confounders) and in the analysis (to account for the variability and sometimes to reduce bias). For example, nuisance variables can be used as blocking factors or covariates.

**Null and alternative hypotheses:** The null hypothesis (H_0_) is that there is no effect, such as a difference between groups or an association between variables. The alternative hypothesis (H_1_) postulates that an effect exists.

**Outcome measure:** Any variable recorded during a study to assess the effects of a treatment or experimental intervention. Also known as dependent variable, response variable.

**Power:** For a predefined, biologically meaningful effect size, the probability that the statistical test will detect the effect if it exists (i.e., the null hypothesis is rejected correctly).

**Sample size:** Number of experimental units per group, also referred to as *n*.

Definitions are adapted from [14,15] and placed in the context of animal research.

## ARRIVE Essential 10

The ARRIVE Essential 10 (Box 2) constitute the minimum reporting requirement to ensure that reviewers and readers can assess the reliability of the findings presented. There is no ranking within the set; items are presented in a logical order.

**Box 2. ARRIVE Essential 10**

1. Study design
2. Sample size
3. Inclusion and exclusion criteria
4. Randomisation
5. Blinding
6. Outcome measures
7. Statistical methods
8. Experimental animals
9. Experimental procedures
10. Results

### Item 1. Study design

**For each experiment, provide brief details of study design including**:

**1a**. **The groups being compared, including control groups**. **If no control group has been used, the rationale should be stated**.

**Explanation.** The choice of control or comparator group is dependent on the experimental objective. Negative controls are used to determine whether a difference between groups is caused by the intervention (e.g., wild-type animals versus genetically modified animals, placebo versus active treatment, sham surgery versus surgical intervention). Positive controls can be used to support the interpretation of negative results or determine if an expected effect is detectable.

It may not be necessary to include a separate control with no active treatment if, for example, the experiment aims to compare a treatment administered by different methods (e.g., intraperitoneal administration versus oral gavage) or animals that are used as their own control in a longitudinal study. A pilot study, such as one designed to test the feasibility of a procedure, might also not require a control group.

For complex study designs, a visual representation is more easily interpreted than a text description, so a timeline diagram or flowchart is recommended. Diagrams facilitate the identification of which treatments and procedures were applied to specific animals or groups of animals and at what point in the study these were performed. They also help to communicate complex design features such as whether factors are crossed or nested (hierarchical/multilevel designs), blocking (to reduce unwanted variation, see Item 4. Randomisation), or repeated measurements over time on the same experimental unit (repeated measures designs); see [16–18] for more information on different design types. The Experimental Design Assistant (EDA) is a platform to support researchers in the design of in vivo experiments; it can be used to generate diagrams to represent any type of experimental design [19].

For each experiment performed, clearly report all groups used. Selectively excluding some experimental groups (for example, because the data are inconsistent or conflict with the narrative of the paper) is misleading and should be avoided [20]. Ensure that test groups, comparators, and controls (negative or positive) can be identified easily. State clearly if the same control group was used for multiple experiments or if no control group was used.

**Examples**

**Subitem 1a—Example 1**

‘The DAV1 study is a one-way, two-period crossover trial with 16 piglets receiving amoxicillin and placebo at period 1 and only amoxicillin at period 2. Amoxicillin was administered orally with a single dose of 30 mg.kg^-1^. Plasma amoxicillin concentrations were collected at same sampling times at each period: 0.5, 1, 1.5, 2, 4, 6, 8, 10 and 12 h’ [21].

**Subitem 1a—Example 2**

**Fig 1:**
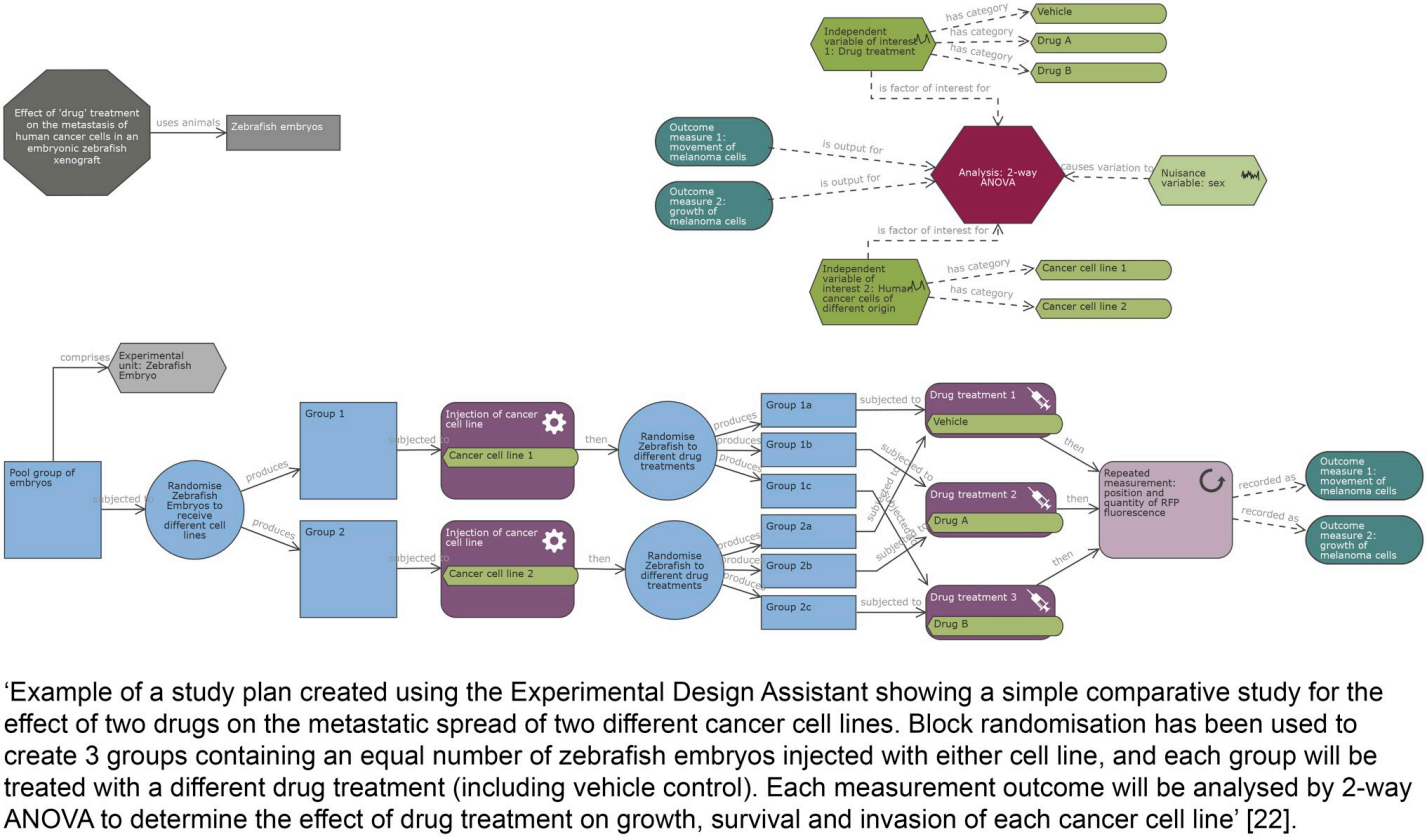
Reproduced from reference [22].

**1b**. **The experimental unit (e.g., a single animal, litter, or cage of animals)**.

**Explanation.** Within a design, biological and technical factors will often be organised hierarchically, such as cells within animals and mitochondria within cells, or cages within rooms and animals within cages. Such hierarchies can make determining the sample size difficult (is it the number of animals, cells, or mitochondria?). The sample size is the number of experimental units per group. The experimental unit is defined as the biological entity subjected to an intervention independently of all other units, such that it is possible to assign any two experimental units to different treatment groups. It is also sometimes called the unit of randomisation. In addition, the experimental units should not influence each other on the outcomes that are measured.

Commonly, the experimental unit is the individual animal, each independently allocated to a treatment group (e.g., a drug administered by injection). However, the experimental unit may be the cage or the litter (e.g., a diet administered to a whole cage, or a treatment administered to a dam and investigated in her pups), or it could be part of the animal (e.g., different drug treatments applied topically to distinct body regions of the same animal). Animals may also serve as their own controls, receiving different treatments separated by washout periods; here, the experimental unit is an animal for a period of time. There may also be multiple experimental units in a single experiment, such as when a treatment is given to a pregnant dam and then the weaned pups are allocated to different diets [23]. See [17,24,25] for further guidance on identifying experimental units.

Conflating experimental units with subsamples or repeated measurements can lead to artificial inflation of the sample size. For example, measurements from 50 individual cells from a single mouse represent *n* = 1 when the experimental unit is the mouse. The 50 measurements are subsamples and provide an estimate of measurement error and so should be averaged or used in a nested analysis. Reporting *n* = 50 in this case is an example of pseudoreplication [26]. It underestimates the true variability in a study, which can lead to false positives and invalidate the analysis and resulting conclusions [26,27]. If, however, each cell taken from the mouse is then randomly allocated to different treatments and assessed individually, the cell might be regarded as the experimental unit.

Clearly indicate the experimental unit for each experiment so that the sample sizes and statistical analyses can be properly evaluated.

**Examples**

**Subitem 1b—Example 1**

‘The present study used the tissues collected at E15.5 from dams fed the 1X choline and 4X choline diets (*n* = 3 dams per group, per fetal sex; total *n* = 12 dams). To ensure statistical independence, only one placenta (either male or female) from each dam was used for each experiment. Each placenta, therefore, was considered to be an experimental unit’ [28].

**Subitem 1b—Example 2**

‘We have used data collected from high-throughput phenotyping, which is based on a pipeline concept where a mouse is characterized by a series of standardized and validated tests underpinned by standard operating procedures (SOPs)…. The individual mouse was considered the experimental unit within the studies’ [29].

**Subitem 1b—Example 3**

‘Fish were divided in two groups according to weight (0.7–1.2 g and 1.3–1.7 g) and randomly stocked (at a density of 15 fish per experimental unit) in 24 plastic tanks holding 60 L of water’ [30].

**Subitem 1b—Example 4**

‘In the study, *n* refers to number of animals, with five acquisitions from each [corticostriatal] slice, with a maximum of three slices obtained from each experimental animal used for each protocol (six animals each group)’ [31].

### Item 2. Sample size

**2a**. **Specify the exact number of experimental units allocated to each group, and the total number in each experiment**. **Also indicate the total number of animals used**.

**Explanation.** The sample size relates to the number of experimental units in each group at the start of the study and is usually represented by *n* (see Item 1. Study design for further guidance on identifying and reporting experimental units). This information is crucial to assess the validity of the statistical model and the robustness of the experimental results.

The sample size in each group at the start of the study may be different from the *n* numbers in the analysis (see Item 3. Inclusion and exclusion criteria); this information helps readers identify attrition or if there have been exclusions and in which group they occurred. Reporting the total number of animals used in the study is also useful to identify whether any were reused between experiments.

Report the exact value of *n* per group and the total number in each experiment (including any independent replications). If the experimental unit is not the animal, also report the total number of animals to help readers understand the study design. For example, in a study investigating diet using cages of animals housed in pairs, the number of animals is double the number of experimental units.

**Example**

**Subitem 2a –example 1**

**Fig 2:**
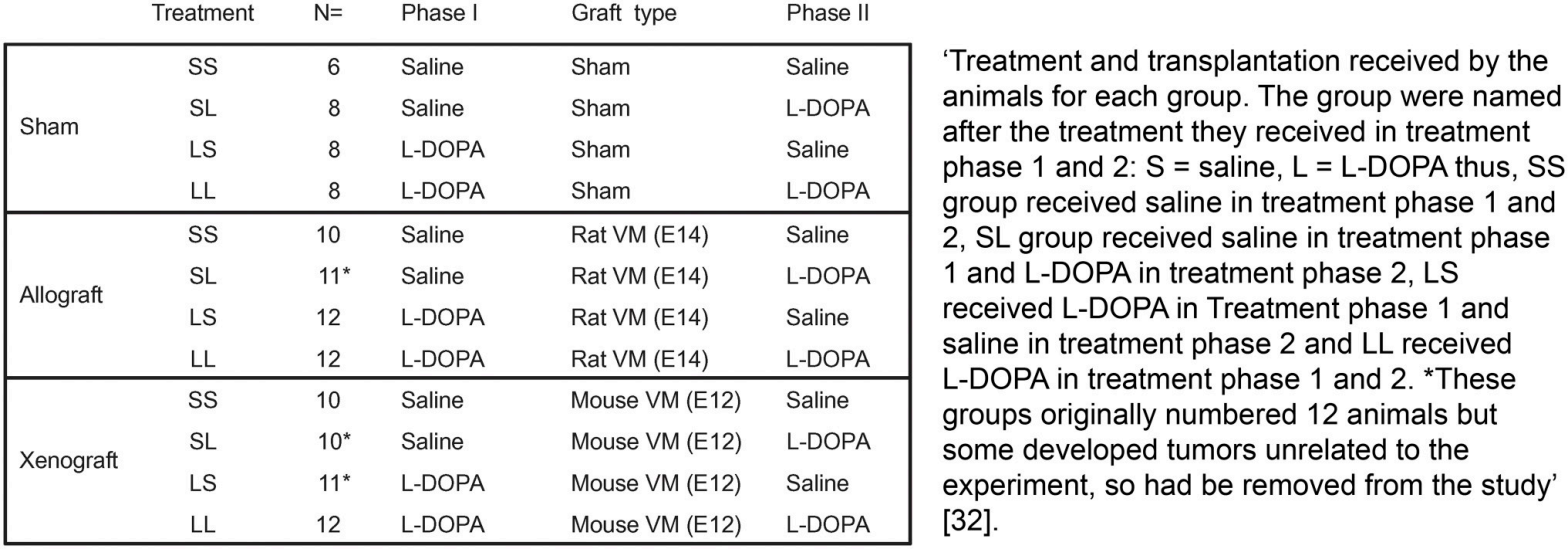
Reproduced from reference [32].

**2b**. **Explain how the sample size was decided**. **Provide details of any a priori sample size calculation, if done**.

**Explanation.** For any type of experiment, it is crucial to explain how the sample size was determined. For hypothesis-testing experiments, in which inferential statistics are used to estimate the size of the effect and to determine the weight of evidence against the null hypothesis, the sample size needs to be justified to ensure experiments are of an optimal size to test the research question [33,34] (see Item 13. Objectives). Sample sizes that are too small (i.e., underpowered studies) produce inconclusive results, whereas sample sizes that are too large (i.e., overpowered studies) raise ethical issues over unnecessary use of animals and may produce trivial findings that are statistically significant but not biologically relevant [35]. Low power has three effects: first, within the experiment, real effects are more likely to be missed; second, when an effect is detected, this will often be an overestimation of the true effect size [24]; and finally, when low power is combined with publication bias, there is an increase in the false positive rate in the published literature [36]. Consequently, low-powered studies contribute to the poor internal validity of research and risk wasting animals used in inconclusive research [37].

Study design can influence the statistical power of an experiment, and the power calculation used needs to be appropriate for the design implemented. Statistical programmes to help perform a priori sample size calculations exist for a variety of experimental designs and statistical analyses, both freeware (web-based applets and functions in R) and commercial software [38–40]. Choosing the appropriate calculator or algorithm to use depends on the type of outcome measures and independent variables, and the number of groups. Consultation with a statistician is recommended, especially when the experimental design is complex or unusual.

When the experiment tests the effect of an intervention on the mean of a continuous outcome measure, the sample size can be calculated a priori, based on a mathematical relationship between the predefined, biologically relevant effect size, variability estimated from prior data, chosen significance level, power, and sample size (see Box 3 and [17,41] for practical advice). If you have used an a priori sample size calculation, report

- the analysis method (e.g., two-tailed Student *t* test with a 0.05 significance threshold)
- the effect size of interest and a justification explaining why an effect size of that magnitude is relevant
- the estimate of variability used (e.g., standard deviation) and how it was estimated
- the power selected

**Box 3. Information used in a power calculation**

Sample size calculation is based on a mathematical relationship between the following parameters: effect size, variability, significance level, power, and sample size. Questions to consider are the following:

**The primary objective of the experiment—What is the main outcome measure?**

The primary outcome measure should be identified in the planning stage of the experiment; it is the outcome of greatest importance, which will answer the main experimental question.

**The predefined effect size—What is a biologically relevant effect size?**

The effect size is estimated as a biologically relevant change in the primary outcome measure between the groups under study. This can be informed by similar studies and involves scientists exploring what magnitude of effect would generate interest and would be worth taking forward into further work. In preclinical studies, the clinical relevance of the effect should also be taken into consideration.

**What is the estimate of variability?**

Estimates of variability can be obtained

- From data collected from a preliminary experiment conducted under identical conditions to the planned experiment, e.g., a previous experiment in the same laboratory, testing the same treatment under similar conditions on animals with the same characteristics
- From the control group in a previous experiment testing a different treatment
- From a similar experiment reported in the literature

**Significance threshold—What risk of a false positive is acceptable?**

The significance level or threshold (α) is the probability of obtaining a false positive. If it is set at 0.05, then the risk of obtaining a false positive is 1 in 20 for a single statistical test. However, the threshold or the *p*-values will need to be adjusted in scenarios of multiple testing (e.g., by using a Bonferroni correction).

**Power—What risk of a false negative is acceptable?**

For a predefined, biologically meaningful effect size, the power (1 − β) is the probability that the statistical test will detect the effect if it genuinely exists (i.e., true positive result). A target power between 80% and 95% is normally deemed acceptable, which entails a risk of false negative between 5% and 20%.

**Directionality—Will you use a one- or two-sided test?**

The directionality of a test depends on the distribution of the test statistics for a given analysis. For tests based on *t* or *z* distributions (such as *t* tests), whether the data will be analysed using a one- or two-sided test relates to whether the alternative hypothesis is directional or not. An experiment with a directional (one-sided) alternative hypothesis can be powered and analysed with a one-sided test with the goal of maximising the sensitivity to detect this directional effect. Controversy exists within the statistics community on when it is appropriate to use a one-sided test [42]. The use of a one-sided test requires justification of why a treatment effect is only of interest when it is in a defined direction and why they would treat a large effect in the unexpected direction no differently from a nonsignificant difference [43]. Following the use of a one-sided test, the investigator cannot then test for the possibility of missing an effect in the untested direction. Choosing a one-tailed test for the sole purpose of attaining statistical significance is not appropriate.

Two-sided tests with a nondirectional alternative hypothesis are much more common and allow researchers to detect the effect of a treatment regardless of its direction.

Note that analyses such as ANOVA and chi-squared are based on asymmetrical distributions (F-distribution and chi-squared distribution) with only one tail. Therefore, these tests do not have a directionality option.

There are several types of studies in which a priori sample size calculations are not appropriate. For example, the number of animals needed for antibody or tissue production is determined by the amount required and the production ability of an individual animal. For studies in which the outcome is the successful generation of a sample or a condition (e.g., the production of transgenic animals), the number of animals is determined by the probability of success of the experimental procedure.

In early feasibility or pilot studies, the number of animals required depends on the purpose of the study. When the objective of the preliminary study is primarily logistic or operational (e.g., to improve procedures and equipment), the number of animals needed is generally small. In such cases, power calculations are not appropriate and sample sizes can be estimated based on operational capacity and constraints [44]. Pilot studies alone are unlikely to provide adequate data on variability for a power calculation for future experiments. Systematic reviews and previous studies are more appropriate sources of information on variability [45].

If no power calculation was used to determine the sample size, state this explicitly and provide the reasoning that was used to decide on the sample size per group. Regardless of whether a power calculation was used or not, when explaining how the sample size was determined take into consideration any anticipated loss of animals or data, for example, due to exclusion criteria established upfront or expected attrition (see Item 3. Inclusion and exclusion criteria).

**Examples**

**Subitem 2b—Example 1**

‘The sample size calculation was based on postoperative pain numerical rating scale (NRS) scores after administration of buprenorphine (NRS AUC mean = 2.70; noninferiority limit = 0.54; standard deviation = 0.66) as the reference treatment… and also Glasgow Composite Pain Scale (GCPS) scores… using online software (Experimental design assistant; https://eda.nc3rs.org.uk/eda/login/auth). The power of the experiment was set to 80%. A total of 20 dogs per group were considered necessary’ [46].

**Subitem 2b—Example 2**

‘We selected a small sample size because the bioglass prototype was evaluated in vivo for the first time in the present study, and therefore, the initial intention was to gather basic evidence regarding the use of this biomaterial in more complex experimental designs’ [47].

### Item 3. Inclusion and exclusion criteria

**3a**. **Describe any criteria used for including or excluding animals (or experimental units) during the experiment, and data points during the analysis**. **Specify if these criteria were established a priori**. **If no criteria were set, state this explicitly**.

**Explanation.** Inclusion and exclusion criteria define the eligibility or disqualification of animals and data once the study has commenced. To ensure scientific rigour, the criteria should be defined before the experiment starts and data are collected [8,33,48,49]. Inclusion criteria should not be confused with animal characteristics (see Item 8. Experimental animals) but can be related to these (e.g., body weights must be within a certain range for a particular procedure) or related to other study parameters (e.g., task performance has to exceed a given threshold). In studies in which selected data are reanalysed for a different purpose, inclusion and exclusion criteria should describe how data were selected.

Exclusion criteria may result from technical or welfare issues such as complications anticipated during surgery or circumstances in which test procedures might be compromised (e.g., development of motor impairments that could affect behavioural measurements). Criteria for excluding samples or data include failure to meet quality control standards, such as insufficient sample volumes, unacceptable levels of contaminants, poor histological quality, etc. Similarly, how the researcher will define and handle data outliers during the analysis should also be decided before the experiment starts (see subitem 3b for guidance on responsible data cleaning).

Exclusion criteria may also reflect the ethical principles of a study in line with its humane endpoints (see Item 16. Animal care and monitoring). For example, in cancer studies, an animal might be dropped from the study and euthanised before the predetermined time point if the size of a subcutaneous tumour exceeds a specific volume [50]. If losses are anticipated, these should be considered when determining the number of animals to include in the study (see Item 2. Sample size). Whereas exclusion criteria and humane endpoints are typically included in the ethical review application, reporting the criteria used to exclude animals or data points in the manuscript helps readers with the interpretation of the data and provides crucial information to other researchers wanting to adopt the model.

Best practice is to include all a priori inclusion and exclusion/outlier criteria in a preregistered protocol (see Item 19. Protocol registration). At the very least, these criteria should be documented in a laboratory notebook and reported in manuscripts, explicitly stating that the criteria were defined before any data was collected.

**Example**

**Subitem 3a—Example 1**

‘The animals were included in the study if they underwent successful MCA occlusion (MCAo), defined by a 60% or greater drop in cerebral blood flow seen with laser Doppler flowmetry. The animals were excluded if insertion of the thread resulted in perforation of the vessel wall (determined by the presence of sub-arachnoid blood at the time of sacrifice), if the silicon tip of the thread became dislodged during withdrawal, or if the animal died prematurely, preventing the collection of behavioral and histological data’ [51].

**3b**. **For each experimental group, report any animals, experimental units, or data points not included in the analysis and explain why**. **If there were no exclusions, state so**.

**Explanation.** Animals, experimental units, or data points that are unaccounted for can lead to instances in which conclusions cannot be supported by the raw data [52]. Reporting exclusions and attritions provides valuable information to other investigators evaluating the results or who intend to repeat the experiment or test the intervention in other species. It may also provide important safety information for human trials (e.g., exclusions related to adverse effects).

There are many legitimate reasons for experimental attrition, some of which are anticipated and controlled for in advance (see subitem 3a on defining exclusion and inclusion criteria), but some data loss might not be anticipated. For example, data points may be excluded from analyses because of an animal receiving the wrong treatment, unexpected drug toxicity, infections or diseases unrelated to the experiment, sampling errors (e.g., a malfunctioning assay that produced a spurious result, inadequate calibration of equipment), or other human error (e.g., forgetting to switch on equipment for a recording).

Most statistical analysis methods are extremely sensitive to outliers and missing data. In some instances, it may be scientifically justifiable to remove outlying data points from an analysis, such as obvious errors in data entry or measurement with readings that are outside a plausible range. Inappropriate data cleaning has the potential to bias study outcomes [53]; providing the reasoning for removing data points enables the distinction to be made between responsible data cleaning and data manipulation. Missing data, common in all areas of research, can impact the sensitivity of the study and also lead to biased estimates, distorted power, and loss of information if the missing values are not random [54]. Analysis plans should include methods to explore why data are missing. It is also important to consider and justify analysis methods that account for missing data [55,56].

There is a movement toward greater data sharing (see Item 20. Data access), along with an increase in strategies such as code sharing to enable analysis replication. These practices, however transparent, still need to be accompanied by a disclosure on the reasoning for data cleaning and whether methods were defined before any data were collected.

Report all animal exclusions and loss of data points, along with the rationale for their exclusion. For example, this information can be summarised as a table or a flowchart describing attrition in each treatment group. Accompanying this information should be an explicit description of whether researchers were blinded to the group allocations when data or animals were excluded (see Item 5. Blinding and [57]). Explicitly state when built-in models in statistics packages have been used to remove outliers (e.g., GraphPad Prism’s outlier test).

**Examples**

**Subitem 3b—Example 1**

‘Pen was the experimental unit for all data. One entire pen (ZnAA90) was removed as an outlier from both Pre-RAC and RAC periods for poor performance caused by illness unrelated to treatment…. Outliers were determined using Cook’s D statistic and removed if Cook’s D > 0.5. One steer was determined to be an outlier for day 48 liver biopsy TM and data were removed’ [58].

**Subitem 3b—Example 2**

‘Seventy-two SHRs were randomized into the study, of which 13 did not meet our inclusion and exclusion criteria because the drop in cerebral blood flow at occlusion did not reach 60% (seven animals), postoperative death (one animal: autopsy unable to identify the cause of death), haemorrhage during thread insertion (one animal), and disconnection of the silicon tip of the thread during withdrawal, making the permanence of reperfusion uncertain (four animals). A total of 59 animals were therefore included in the analysis of infarct volume in this study. In error, three animals were sacrificed before their final assessment of neurobehavioral score: one from the normothermia/water group and two from the hypothermia/pethidine group. These errors occurred blinded to treatment group allocation. A total of 56 animals were therefore included in the analysis of neurobehavioral score’ [51].

**Subitem 3b—Example 3**

**Fig 3:**
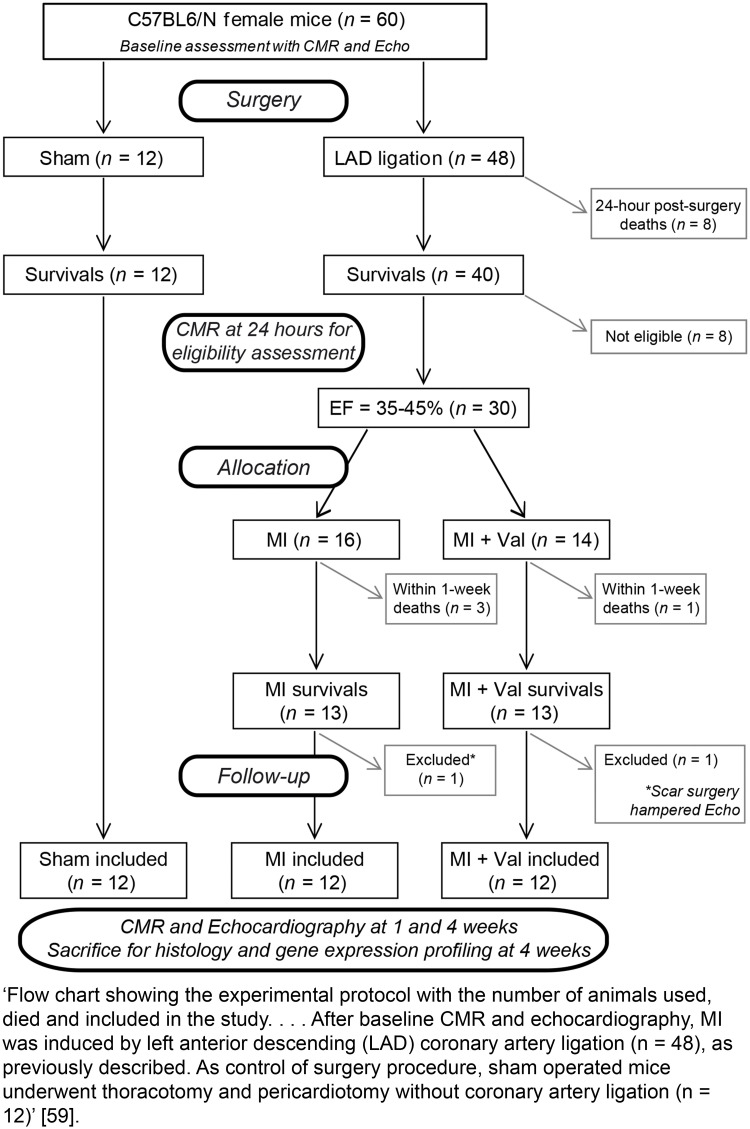
Reproduced from reference [59].

**3c**. **For each analysis, report the exact value of *n* in each experimental group**.

**Explanation.** The exact number of experimental units analysed in each group (i.e., the *n* number) is essential information for the reader to interpret the analysis; it should be reported unambiguously. All animals and data used in the experiment should be accounted for in the data presented. Sometimes, for good reasons, animals may need to be excluded from a study (e.g., illness or mortality), or data points excluded from analyses (e.g., biologically implausible values). Reporting losses will help the reader to understand the experimental design process, replicate methods, and provide adequate tracking of animal numbers in a study, especially when sample size numbers in the analyses do not match the original group numbers.

For each outcome measure, indicate numbers clearly within the text or on figures and provide absolute numbers (e.g., 10/20, not 50%). For studies in which animals are measured at different time points, explicitly report the full description of which animals undergo measurement and when [33].

**Examples**

**Subitem 3c—Example 1**

‘Group F contained 29 adult males and 58 adult females in 2010 (*n* = 87), and 32 adult males and 66 adult females in 2011 (*n* = 98). The increase in female numbers was due to maturation of juveniles to adults. Females belonged to three matrilines, and there were no major shifts in rank in the male hierarchy. Six mid to low ranking individuals died and were excluded from analyses, as were five mid-ranking males who emigrated from the group at the beginning of 2011’ [60].

**Subitem 3c—Example 2**

‘The proportion of test time that animals spent interacting with the handler (sniffed the gloved hand or tunnel, made paw contact, climbed on, or entered the handling tunnel) was measured from DVD recordings. This was then averaged across the two mice in each cage as they were tested together and their behaviour was not independent…. Mice handled with the home cage tunnel spent a much greater proportion of the test interacting with the handler (mean ± s.e.m., 39.8 ± 5.2 percent time of 60 s test, n = 8 cages) than those handled by tail (6.4 ± 2.0 percent time, n = 8 cages), while those handled by cupping showed intermediate levels of voluntary interaction (27.6 ± 7.1 percent time, n = 8 cages)’ [61].

### Item 4. Randomisation

**4a**. **State whether randomisation was used to allocate experimental units to control and treatment groups**. **If done, provide the method used to generate the randomisation sequence**.

**Explanation.** Using appropriate randomisation methods during the allocation to groups ensures that each experimental unit has an equal probability of receiving a particular treatment and provides balanced numbers in each treatment group. Selecting an animal ‘at random’ (i.e., haphazardly or arbitrarily) from a cage is not statistically random, as the process involves human judgement. It can introduce bias that influences the results, as a researcher may (consciously or subconsciously) make judgements in allocating an animal to a particular group, or because of unknown and uncontrolled differences in the experimental conditions or animals in different groups. Using a validated method of randomisation helps minimise selection bias and reduce systematic differences in the characteristics of animals allocated to different groups [62–64]. Inferential statistics based on nonrandomised group allocation are not valid [65,66]. Thus, the use of randomisation is a prerequisite for any experiment designed to test a hypothesis. Examples of appropriate randomisation methods include online random number generators (e.g., https://www.graphpad.com/quickcalcs/randomize1/) or a function like Rand() in spreadsheet software such as Excel, Google Sheets, or LibreOffice. The EDA has a dedicated feature for randomisation and allocation concealment [19].

Systematic reviews have shown that animal experiments that do not report randomisation or other bias-reducing measures such as blinding are more likely to report exaggerated effects that meet conventional measures of statistical significance [67–69]. It is especially important to use randomisation in situations in which it is not possible to blind all or parts of the experiment, but even with randomisation, researcher bias can pervert the allocation. This can be avoided by using allocation concealment (see Item 5. Blinding). In studies in which sample sizes are small, simple randomisation may result in unbalanced groups; here, randomisation strategies to balance groups such as randomising in matched pairs [70–72] and blocking are encouraged [17]. Reporting the precise method used to allocate animals or experimental units to groups enables readers to assess the reliability of the results and identify potential limitations.

Report the type of randomisation used (simple, stratified, randomised complete blocks, etc.; see Box 4), the method used to generate the randomisation sequence (e.g., computer-generated randomisation sequence, with details of the algorithm or programme used), and what was randomised (e.g., treatment to experimental unit, order of treatment for each animal). If this varies between experiments, report this information specifically for each experiment. If randomisation was not the method used to allocate experimental units to groups, state this explicitly and explain how the groups being compared were formed.

**Box 4. Considerations for the randomisation strategy**

**Simple randomisation**

All animals/samples are simultaneously randomised to the treatment groups without considering any other variable. This strategy is rarely appropriate, as it cannot ensure that comparison groups are balanced for other variables that might influence the result of an experiment.

**Randomisation within blocks**

Blocking is a method of controlling natural variation among experimental units. This splits up the experiment into smaller subexperiments (blocks), and treatments are randomised to experimental units within each block [17,66,73]. This takes into account nuisance variables that could potentially bias the results (e.g., cage location, day or week of procedure).

Stratified randomisation uses the same principle as randomisation within blocks, only the strata tend to be traits of the animal that are likely to be associated with the response (e.g., weight class or tumour size class). This can lead to differences in the practical implementation of stratified randomisation as compared with block randomisation (e.g., there may not be equal numbers of experimental units in each weight class).

**Other randomisation strategies**

Minimisation is an alternative strategy to allocate animals/samples to treatment group to balance variables that might influence the result of an experiment. With minimisation, the treatment allocated to the next animal/sample depends on the characteristics of those animals/samples already assigned. The aim is that each allocation should minimise the imbalance across multiple factors [74]. This approach works well for a continuous nuisance variable such as body weight or starting tumour volume.

**Examples of nuisance variables that can be accounted for in the randomisation strategy**

- Time or day of the experiment
- Litter, cage, or fish tank
- Investigator or surgeon—different level of experience in the people administering the treatments, performing the surgeries, or assessing the results may result in varying stress levels in the animals or duration of anaesthesia
- Equipment (e.g., PCR machine, spectrophotometer)—calibration may vary
- Measurement of a study parameter (e.g., initial tumour volume)
- Animal characteristics (e.g., sex, age bracket, weight bracket)
- Location—exposure to light, ventilation, and disturbances may vary in cages located at different height or on different racks, which may affect important physiological processes

**Implication for the analysis**

If blocking factors are used in the randomisation, they should also be included in the analysis. Nuisance variables increase variability in the sample, which reduces statistical power. Including a nuisance variable as a blocking factor in the analysis accounts for that variability and can increase the power, thus increasing the ability to detect a real effect with fewer experimental units. However, blocking uses up degrees of freedom and thus reduces the power if the nuisance variable does not have a substantial impact on variability.

**Examples**

**Subitem 4a—Example 1**

‘Fifty 12-week-old male Sprague-Dawley rats, weighing 320–360g, were obtained from Guangdong Medical Laboratory Animal Center (Guangzhou, China) and randomly divided into two groups (25 rats/group): the intact group and the castration group. Random numbers were generated using the standard = RAND() function in Microsoft Excel’ [75].

**Subitem 4a—Example 2**

‘Animals were randomized after surviving the initial I/R, using a computer based random order generator’ [76].

**Subitem 4a—Example 3**

‘At each institute, phenotyping data from both sexes is collected at regular intervals on age-matched wildtype mice of equivalent genetic backgrounds. Cohorts of at least seven homozygote mice of each sex per pipeline were generated…. The random allocation of mice to experimental group (wildtype versus knockout) was driven by Mendelian Inheritance’ [29].

**4b**. **Describe the strategy used to minimise potential confounders such as the order of treatments and measurements, or animal/cage location**. **If confounders were not controlled, state this explicitly**.

**Explanation.** Ensuring there is no systematic difference between animals in different groups apart from the experimental exposure is an important principle throughout the conduct of the experiment. Identifying nuisance variables (sources of variability or conditions that could potentially bias results) and managing them in the design and analysis increases the sensitivity of the experiment. For example, rodents in cages at the top of the rack may be exposed to higher light levels, which can affect stress [77].

Reporting the strategies implemented to minimise potential differences that arise between treatment groups during the course of the experiment enables others to assess the internal validity. Strategies to report include standardising (keeping conditions the same, e.g., all surgeries done by the same surgeon), randomising (e.g., the sampling or measurement order), and blocking or counterbalancing (e.g., position of animal cages or tanks on the rack), to ensure groups are similarly affected by a source of variability. In some cases, practical constraints prevent some nuisance variables from being randomised, but they can still be accounted for in the analysis (see Item 7. Statistical methods).

Report the methods used to minimise confounding factors alongside the methods used to allocate animals to groups. If no measures were used to minimise confounders (e.g., treatment order, measurement order, cage or tank position on a rack), explicitly state this and explain why.

**Examples**

**Subitem 4b—Example 1**

‘Randomisation was carried out as follows. On arrival from El-Nile Company, animals were assigned a group designation and weighed. A total number of 32 animals were divided into four different weight groups (eight animals per group). Each animal was assigned a temporary random number within the weight range group. On the basis of their position on the rack, cages were given a numerical designation. For each group, a cage was selected randomly from the pool of all cages. Two animals were removed from each weight range group and given their permanent numerical designation in the cages. Then, the cages were randomized within the exposure group’ [78].

**Subitem 4b—Example 2**

‘… test time was between 08.30am to 12.30pm and testing order was randomized daily, with each animal tested at a different time each test day’ [79].

**Subitem 4b—Example 3**

‘Bulls were blocked by BW into four blocks of 905 animals with similar BW and then within each block, bulls were randomly assigned to one of four experimental treatments in a completely randomized block design resulting in 905 animals per treatment. Animals were allocated to 20 pens (181 animals per pen and five pens per treatment)’ [80].

### Item 5. Blinding

**Describe who was aware of the group allocation at the different stages of the experiment (during the allocation, the conduct of the experiment, the outcome assessment, and the data analysis)**.

**Explanation.** Researchers often expect a particular outcome and can unintentionally influence the experiment or interpret the data in such a way as to support their preferred hypothesis [81]. Blinding is a strategy used to minimise these subjective biases.

Although there is primary evidence of the impact of blinding in the clinical literature that directly compares blinded versus unblinded assessment of outcomes [82], there is limited empirical evidence in animal research [83,84]. There are, however, compelling data from systematic reviews showing that nonblinded outcome assessment leads to the treatment effects being overestimated, and the lack of bias-reducing measures such as randomisation and blinding can contribute to as much as 30%–45% inflation of effect sizes [67,68,85].

Ideally, investigators should be unaware of the treatment(s) animals have received or will be receiving, from the start of the experiment until the data have been analysed. If this is not possible for every stage of an experiment (see Box 5), it should always be possible to conduct at least some of the stages blind. This has implications for the organisation of the experiment and may require help from additional personnel—for example, a surgeon to perform interventions, a technician to code the treatment syringes for each animal, or a colleague to code the treatment groups for the analysis. Online resources are available to facilitate allocation concealment and blinding [19].

**Box 5. Blinding during different stages of an experiment**

**During allocation**

Allocation concealment refers to concealing the treatment to be allocated to each individual animal from those assigning the animals to groups, until the time of assignment. Together with randomisation, allocation concealment helps minimise selection bias, which can introduce systematic differences between treatment groups.

**During the conduct of the experiment**

When possible, animal care staff and those who administer treatments should be unaware of allocation groups to ensure that all animals in the experiment are handled, monitored, and treated in the same way. Treating different groups differently based on the treatment they have received could alter animal behaviour and physiology and produce confounds.

Welfare or safety reasons may prevent blinding of animal care staff, but in most cases, blinding is possible. For example, if hazardous microorganisms are used, control animals can be considered as dangerous as infected animals. If a welfare issue would only be tolerated for a short time in treated but not control animals, a harm-benefit analysis is needed to decide whether blinding should be used.

**During the outcome assessment**

The person collecting experimental measurements or conducting assessments should not know which treatment each sample/animal received and which samples/animals are grouped together. Blinding is especially important during outcome assessment, particularly if there is a subjective element (e.g., when assessing behavioural changes or reading histological slides) [83]. Randomising the order of examination can also reduce bias.

If the person assessing the outcome cannot be blinded to the group allocation (e.g., obvious phenotypic or behavioural differences between groups), some, but not all, of the sources of bias could be mitigated by sending data for analysis to a third party who has no vested interest in the experiment and does not know whether a treatment is expected to improve or worsen the outcome.

**During the data analysis**

The person analysing the data should know which data are grouped together to enable group comparisons but should not be aware of which specific treatment each group received. This type of blinding is often neglected but is important, as the analyst makes many semisubjective decisions such as applying data transformation to outcome measures, choosing methods for handling missing data, and handling outliers. How these decisions will be made should also be decided a priori.

Data can be coded prior to analysis so that the treatment group cannot be identified before analysis is completed.

Specify whether blinding was used or not for each step of the experimental process (see Box 5) and indicate what particular treatment or condition the investigators were blinded to, or aware of.

If blinding was not used at any of the steps outlined in Box 5, explicitly state this and provide the reason why blinding was not possible or not considered.

**Examples**

**Item 5—Example 1**

‘For each animal, four different investigators were involved as follows: a first investigator (RB) administered the treatment based on the randomization table. This investigator was the only person aware of the treatment group allocation. A second investigator (SC) was responsible for the anaesthetic procedure, whereas a third investigator (MS, PG, IT) performed the surgical procedure. Finally, a fourth investigator (MAD) (also unaware of treatment) assessed GCPS and NRS, mechanical nociceptive threshold (MNT), and sedation NRS scores’ [46].

**Item 5—Example 2**

‘… due to overt behavioral seizure activity the experimenter could not be blinded to whether the animal was injected with pilocarpine or with saline’ [86].

**Item 5—Example 3**

‘Investigators could not be blinded to the mouse strain due to the difference in coat colors, but the three-chamber sociability test was performed with ANY-maze video tracking software (Stoelting, Wood Dale, IL, USA) using an overhead video camera system to automate behavioral testing and provide unbiased data analyses. The one-chamber social interaction test requires manual scoring and was analyzed by an individual with no knowledge of the questions’ [87].

### Item 6. Outcome measures

**6a**. **Clearly define all outcome measures assessed (e.g., cell death, molecular markers, or behavioural changes)**.

**Explanation.** An outcome measure (also known as a dependent variable or a response variable) is any variable recorded during a study (e.g., volume of damaged tissue, number of dead cells, specific molecular marker) to assess the effects of a treatment or experimental intervention. Outcome measures may be important for characterising a sample (e.g., baseline data) or for describing complex responses (e.g., ‘haemodynamic’ outcome measures including heart rate, blood pressure, central venous pressure, and cardiac output). Failure to disclose all the outcomes that were measured introduces bias in the literature, as positive outcomes (e.g., those statistically significant) are reported more often [88–91].

Explicitly describe what was measured, especially when measures can be operationalised in different ways. For example, activity could be recorded as time spent moving or distance travelled. When possible, the recording of outcome measures should be made in an unbiased manner (e.g., blinded to the treatment allocation of each experimental group; see Item 5. Blinding). Specify how the outcome measure(s) assessed are relevant to the objectives of the study.

**Example**

**Subitem 6a—Example 1**

‘The following parameters were assessed: threshold pressure (TP; intravesical pressure immediately before micturition); post-void pressure (PVP; intravesical pressure immediately after micturition); peak pressure (PP; highest intravesical pressure during micturition); capacity (CP; volume of saline needed to induce the first micturition); compliance (CO; CP to TP ratio); frequency of voiding contractions (VC) and frequency of non-voiding contractions (NVCs)’ [92].

**6b**. **For hypothesis-testing studies, specify the primary outcome measure, i.e., the outcome measure that was used to determine the sample size**.

**Explanation.** In a hypothesis-testing experiment, the primary outcome measure answers the main biological question. It is the outcome of greatest importance, identified in the planning stages of the experiment and used as the basis for the sample size calculation (see Box 3). For exploratory studies, it is not necessary to identify a single primary outcome, and often multiple outcomes are assessed (see Item 13. Objectives).

In a hypothesis-testing study powered to detect an effect on the primary outcome measure, data on secondary outcomes are used to evaluate additional effects of the intervention, but subsequent statistical analysis of secondary outcome measures may be underpowered, making results and interpretation less reliable [88,93]. Studies that claim to test a hypothesis but do not specify a predefined primary outcome measure or those that change the primary outcome measure after data were collected (also known as primary outcome switching) are liable to selectively report only statistically significant results, favouring more positive findings [94].

Registering a protocol in advance protects the researcher against concerns about selective outcome reporting (also known as data dredging or p-hacking) and provides evidence that the primary outcome reported in the manuscript accurately reflects what was planned [95] (see Item 19. Protocol registration).

In studies using inferential statistics to test a hypothesis (e.g., *t* test, ANOVA), if more than one outcome was assessed, explicitly identify the primary outcome measure, state whether it was defined as such prior to data collection and whether it was used in the sample size calculation. If there was no primary outcome measure, explicitly state so.

**Examples**

**Subitem 6b—Example 1**

‘The primary outcome of this study will be forelimb function assessed with the staircase test. Secondary outcomes constitute Rotarod performance, stroke volume (quantified on MR imaging or brain sections, respectively), diffusion tensor imaging (DTI) connectome mapping, and histological analyses to measure neuronal and microglial densities, and phagocytic activity…. The study is designed with 80% power to detect a relative 25% difference in pellet-reaching performance in the Staircase test’ [96].

**Subitem 6b—Example 2**

‘The primary endpoint of this study was defined as left ventricular ejection fraction (EF) at the end of follow-up, measured by magnetic resonance imaging (MRI). Secondary endpoints were left ventricular end diastolic volume and left ventricular end systolic volume (EDV and ESV) measured by MRI, infarct size measured by ex vivo gross macroscopy after incubation with triphenyltetrazolium chloride (TTC) and late gadolinium enhancement (LGE) MRI, functional parameters serially measured by pressure volume (PV-)loop and echocardiography, coronary microvascular function by intracoronary pressure- and flow measurements and vascular density and fibrosis on histology’ [76].

### Item 7. Statistical methods

**7a**. **Provide details of the statistical methods used for each analysis, including software used**.

**Explanation.** The statistical analysis methods implemented will reflect the goals and the design of the experiment; they should be decided in advance before data are collected (see Item 19. Protocol registration). Both exploratory and hypothesis-testing studies might use descriptive statistics to summarise the data (e.g., mean and SD, or median and range). In exploratory studies in which no specific hypothesis was tested, reporting descriptive statistics is important for generating new hypotheses that may be tested in subsequent experiments, but it does not allow conclusions beyond the data. In addition to descriptive statistics, hypothesis-testing studies might use inferential statistics to test a specific hypothesis.

Reporting the analysis methods in detail is essential to ensure readers and peer reviewers can assess the appropriateness of the methods selected and judge the validity of the output. The description of the statistical analysis should provide enough detail so that another researcher could reanalyse the raw data using the same method and obtain the same results. Make it clear which method was used for which analysis.

Analysing the data using different methods and selectively reporting those with statistically significant results constitutes p-hacking and introduces bias in the literature [90,94]. Report all analyses performed in full. Relevant information to describe the statistical methods include

- the outcome measures
- the independent variables of interest
- the nuisance variables taken into account in each statistical test (e.g., as blocking factors or covariates)
- what statistical analyses were performed and references for the methods used
- how missing values were handled
- adjustment for multiple comparisons
- the software package and version used, including computer code if available [97]

The outcome measure is potentially affected by the treatments or interventions being tested but also by other factors, such as the properties of the biological samples (sex, litter, age, weight, etc.) and technical considerations (cage, time of day, batch, experimenter, etc.). To reduce the risk of bias, some of these factors can be taken into account in the design of the experiment, for example, by using blocking factors in the randomisation (see Item 4. Randomisation). Factors deemed to affect the variability of the outcome measure should also be handled in the analysis, for example, as a blocking factor (e.g., batch of reagent or experimenter) or as a covariate (e.g., starting tumour size at point of randomisation).

Furthermore, to conduct the analysis appropriately, it is important to recognise the hierarchy that can exist in an experiment. The hierarchy can induce a clustering effect; for example, cage, litter, or animal effects can occur when the outcomes measured for animals from the same cage/litter, or for cells from the same animal, are more similar to each other. This relationship has to be managed in the statistical analysis by including cage/litter/animal effects in the model or by aggregating the outcome measure to the cage/litter/animal level. Thus, describing the reality of the experiment and the hierarchy of the data, along with the measures taken in the design and the analysis to account for this hierarchy, is crucial to assessing whether the statistical methods used are appropriate.

For bespoke analysis—for example, regression analysis with many terms—it is essential to describe the analysis pipeline in detail. This could include detailing the starting model and any model simplification steps.

When reporting descriptive statistics, explicitly state which measure of central tendency is reported (e.g., mean or median) and which measure of variability is reported (e.g., standard deviation, range, quartiles, or interquartile range). Also describe any modification made to the raw data before analysis (e.g., relative quantification of gene expression against a housekeeping gene). For further guidance on statistical reporting, refer to the Statistical Analyses and Methods in the Published Literature (SAMPL) guidelines [98].

**Examples**

**Subitem 7a—Example 1**

‘Analysis of variance was performed using the GLM procedure of SAS (SAS Inst., Cary, NC). Average pen values were used as the experimental unit for the performance parameters. The model considered the effects of block and dietary treatment (5 diets). Data were adjusted by the covariant of initial body weight. Orthogonal contrasts were used to test the effects of SDPP processing (UV vs no UV) and dietary SDPP level (3% vs 6%). Results are presented as least squares means. The level of significance was set at P < 0.05’ [99].

**Subitem 7a—Example 2**

‘All risk factors of interest were investigated in a single model. Logistic regression allows blocking factors and explicitly investigates the effect of each independent variable controlling for the effects of all others…. As we were interested in husbandry and environmental effects, we blocked the analysis by important biological variables (age; backstrain; inbreeding; sex; breeding status) to control for their effect. (The role of these biological variables in barbering behavior, particularly with reference to barbering as a model for the human disorder trichotillomania, is described elsewhere …). We also blocked by room to control for the effect of unknown environmental variables associated with this design variable. We tested for the effect of the following husbandry and environmental risk factors: cage mate relationships (i.e. siblings, non-siblings, or mixed); cage type (i.e. plastic or steel); cage height from floor; cage horizontal position (whether the cage was on the side or the middle of a rack); stocking density; and the number of adults in the cage. Cage material by cage height from floor; and cage material by cage horizontal position interactions were examined, and then removed from the model as they were nonsignificant. N = 1959 mice were included in this analysis’ [100].

**7b**. **Describe any methods used to assess whether the data met the assumptions of the statistical approach, and what was done if the assumptions were not met**.

**Explanation.** Hypothesis tests are based on assumptions about the underlying data. Describing how assumptions were assessed and whether these assumptions are met by the data enables readers to assess the suitability of the statistical approach used. If the assumptions are incorrect, the conclusions may not be valid. For example, the assumptions for data used in parametric tests (such as a *t* test, *z* test, ANOVA, etc.) are that the data are continuous, the residuals from the analysis are normally distributed, the responses are independent, and different groups have similar variances.

There are various tests for normality, for example, the Shapiro-Wilk and Kolmogorov-Smirnov tests. However, these tests have to be used cautiously. If the sample size is small, they will struggle to detect non-normality; if the sample size is large, the tests will detect unimportant deviations. An alternative approach is to evaluate data with visual plots, e.g., normal probability plots, box plots, scatterplots. If the residuals of the analysis are not normally distributed, the assumption may be satisfied using a data transformation in which the same mathematical function is applied to all data points to produce normally distributed data (e.g., log_e_, log_10_, square root).

Other types of outcome measures (binary, categorical, or ordinal) will require different methods of analysis, and each will have different sets of assumptions. For example, categorical data are summarised by counts and percentages or proportions and are analysed by tests of proportions; these analysis methods assume that data are binary, ordinal or nominal, and independent [18].

For each statistical test used (parametric or nonparametric), report the type of outcome measure and the methods used to test the assumptions of the statistical approach. If data were transformed, identify precisely the transformation used and which outcome measures it was applied to. Report any changes to the analysis if the assumptions were not met and an alternative approach was used (e.g., a nonparametric test was used, which does not require the assumption of normality). If the relevant assumptions about the data were not tested, state this explicitly.

**Examples**

**Subitem 7b—Example 1**

‘Model assumptions were checked using the Shapiro-Wilk normality test and Levene’s Test for homogeneity of variance and by visual inspection of residual and fitted value plots. Some of the response variables had to be transformed by applying the natural logarithm or the second or third root, but were back-transformed for visualization of significant effects’ [101].

**Subitem 7b—Example 2**

‘The effects of housing (treatment) and day of euthanasia on cortisol levels were assessed by using fixed-effects 2-way ANOVA. An initial exploratory analysis indicated that groups with higher average cortisol levels also had greater variation in this response variable. To make the variation more uniform, we used a logarithmic transform of each fish’s cortisol per unit weight as the dependent variable in our analyses. This action made the assumptions of normality and homoscedasticity (standard deviations were equal) of our analyses reasonable’ [102].

### Item 8. Experimental animals

**8a**. **Provide species-appropriate details of the animals used, including species, strain and substrain, sex, age or developmental stage, and, if relevant, weight**.

**Explanation.** The species, strain, substrain, sex, weight, and age of animals are critical factors that can influence most experimental results [103–107]. Reporting the characteristics of all animals used is equivalent to standardised human patient demographic data; these data support both the internal and external validity of the study results. It enables other researchers to repeat the experiment and generalise the findings. It also enables readers to assess whether the animal characteristics chosen for the experiment are relevant to the research objectives.

When reporting age and weight, include summary statistics for each experimental group (e.g., mean and standard deviation) and, if possible, baseline values for individual animals (e.g., as supplementary information or a link to a publicly accessible data repository). As body weight might vary during the course of the study, indicate when the measurements were taken. For most species, precise reporting of age is more informative than a description of the developmental status (e.g., a mouse referred to as an adult can vary in age from 6 to 20 weeks [108]). In some cases, however, reporting the developmental stage is more informative than chronological age—for example, in juvenile *Xenopus*, in which rate of development can be manipulated by incubation temperature [109].

Reporting the weight or the sex of the animals used may not feasible for all studies. For example, sex may be unknown for embryos or juveniles, or weight measurement may be particularly stressful for some aquatic species. If reporting these characteristics can be reasonably expected for the species used and the experimental setting but are not reported, provide a justification.

**Examples**

**Subitem 8a—Example 1**

‘One hundred and nineteen male mice were used: C57BL/6OlaHsd mice (*n* = 59), and BALB/c OlaHsd mice (*n* = 60) (both from Harlan, Horst, The Netherlands). At the time of the EPM test the mice were 13 weeks old and had body weights of 27.4 ± 0.4 g and 27.8 ± 0.3 g, respectively (mean ± SEM)’ [110].

**Subitem 8a—Example 2**

‘Histone Methylation Profiles and the Transcriptome of *X*. *tropicalis* Gastrula Embryos. To generate epigenetic profiles, ChIP was performed using specific antibodies against trimethylated H3K4 and H3K27 in *Xenopus* gastrula-stage embryos (Nieuwkoop-Faber stage 11–12), followed by deep sequencing (ChIP-seq). In addition, polyA-selected RNA (stages 10–13) was reverse transcribed and sequenced (RNA-seq)’ [111].

**8b**. **Provide further relevant information on the provenance of animals, health/immune status, genetic modification status, genotype, and any previous procedures**.

**Explanation.** The animals’ provenance, their health or immune status, and their history of previous testing or procedures can influence their physiology and behaviour, as well as their response to treatments, and thus impact on study outcomes. For example, animals of the same strain but from different sources, or animals obtained from the same source but at different times, may be genetically different [16]. The immune or microbiological status of the animals can also influence welfare, experimental variability, and scientific outcomes [112–114].

Report the health status of all animals used in the study and any previous procedures the animals have undergone. For example, if animals are specific pathogen free (SPF), list the pathogens that they were declared free of. If health status is unknown or was not tested, explicitly state this.

For genetically modified animals, describe the genetic modification status (e.g., knockout, overexpression), genotype (e.g., homozygous, heterozygous), manipulated gene(s), genetic methods and technologies used to generate the animals, how the genetic modification was confirmed, and details of animals used as controls (e.g., littermate controls [115]).

Reporting the correct nomenclature is crucial to understanding the data and ensuring that the research is discoverable and replicable [116–118]. Useful resources for reporting nomenclature for different species include

- Mice—International Committee on Standardized Genetic Nomenclature (https://www.jax.org/jax-mice-and-services/customer-support/technical-support/genetics-and-nomenclature)
- Rats—Rat Genome and Nomenclature Committee (https://rgd.mcw.edu/)
- Zebrafish—Zebrafish Information Network (http://zfin.org/)
- *Xenopus*—Xenbase (http://www.xenbase.org/entry/)
- *Drosophila*—FlyBase (http://flybase.org/)
- *C*. *elegans*—WormBase (https://wormbase.org/)

**Examples**

**Subitem 8b—Example 1**

‘A construct was engineered for knockin of the *mi*R-128 (*mi*R-128-3p) gene into the Rosa26 locus. Rosa26 genomic DNA fragments (~1.1 kb and ~4.3 kb 5′ and 3′ homology arms, respectively) were amplified from C57BL/6 BAC DNA, cloned into the pBasicLNeoL vector sequentially by in-fusion cloning, and confirmed by sequencing. The *mi*R-128 gene, under the control of tetO-minimum promoter, was also cloned into the vector between the two homology arms. In addition, the targeting construct also contained a loxP sites flanking the neomycin resistance gene cassette for positive selection and a diphtheria toxin A (DTA) cassette for negative selection. The construct was linearized with ClaI and electroporated into C57BL/6N ES cells. After G418 selection, seven-positive clones were identified from 121 G418-resistant clones by PCR screening. Six-positive clones were expanded and further analyzed by Southern blot analysis, among which four clones were confirmed with correct targeting with single-copy integration. Correctly targeted ES cell clones were injected into blastocysts, and the blastocysts were implanted into pseudo-pregnant mice to generate chimeras by Cyagen Biosciences Inc. Chimeric males were bred with Cre deleted mice from Jackson Laboratories to generate neomycin-free knockin mice. The correct insertion of the *mi*R-128 cassette and successful removal of the neomycin cassette were confirmed by PCR analysis with the primers listed in Supplementary Table… ’ [119].

**Subitem 8b—Example 2**

‘The C57BL/6J (Jackson) mice were supplied by Charles River Laboratories. The C57BL/6JOlaHsd (Harlan) mice were supplied by Harlan. The α-synuclein knockout mice were kindly supplied by Prof…. (Cardiff University, Cardiff, United Kingdom.) and were congenic C57BL/6JCrl (backcrossed for 12 generations). TNFα−/− mice were kindly supplied by Dr…. (Queens University, Belfast, Northern Ireland) and were inbred on a homozygous C57BL/6J strain originally sourced from Bantin & Kingman and generated by targeting C57BL/6 ES cells. T286A mice were obtained from Prof…. (University of California, Los Angeles, CA). These mice were originally congenic C57BL/6J (backcrossed for five generations) and were then inbred (cousin matings) over 14 y, during which time they were outbred with C57BL/6JOlaHsd mice on three separate occasions’ [120].

### Item 9. Experimental procedures

**For each experimental group, including controls, describe the procedures in enough detail to allow others to replicate them, including**:

**9a**. **What was done, how it was done, and what was used**.

**Explanation.** Essential information to describe in the manuscript includes the procedures used to develop the model (e.g., induction of the pathology), the procedures used to measure the outcomes, and pre- and postexperimental procedures, including animal handling, welfare monitoring, and euthanasia. Animal handling can be a source of stress, and the specific method used (e.g., mice picked up by tail or in cupped hands) can affect research outcomes [61,121,122]. Details about animal care and monitoring intrinsic to the procedure are discussed in further detail in Item 16. Animal care and monitoring. Provide enough detail to enable others to replicate the methods and highlight any quality assurance and quality control used [123,124]. A schematic of the experimental procedures with a timeline can give a clear overview of how the study was conducted. Information relevant to distinct types of interventions and resources are described in Table 1.

**Table 1:** Examples of information to include when reporting specific types of experimental procedures and resources.

Procedures	Resources
Pharmacological procedures (intervention and control) Drug formulation Dose Volume Concentration Site and route of administration Frequency of administration Vehicle or carrier solution formulation and volume Any evidence that the pharmacological agent used reaches the target tissue	Cell lines Identification Provenance Verification and authentication RRID [126,127]
Surgical procedures (including sham surgery) Description of the surgical procedure Anaesthetic used (including dose and other information listed in pharmacological procedures section above) Pre- and postanalgesia regimen Presurgery procedures (e.g., fasting) Aseptic techniques Monitoring (e.g., assessment of surgical anaesthetic plane) Whether the procedure is terminal or not Postsurgery procedures Duration of the procedure and duration of anaesthesia Physical variables measured	Reagents (e.g., antibodies, chemicals) Manufacturer Supplier Catalogue number Lot number (if applicable) Purity of the drug (if applicable) RRID
Pathogen infection (intervention and control) Infectious agent Dose load Vehicle or carrier solution formulation and volume Site and route of infection Timing or frequency of infection	Equipment and software Manufacturer Supplier Model/version number Calibration procedures (if applicable) RRID
Euthanasia Method of euthanasia, including the humane standards the method complies with, such as the AVMA [125] Pharmacological agent, if used (including dose and information listed in pharmacological procedures section above) Any measures taken to reduce pain and distress before or during euthanasia Timing of euthanasia Tissues collected post-euthanasia and timing of collection	

AVMA, American Veterinary Medical Association; RRID, Research Resource Identifier.

When available, cite the Research Resource Identifier (RRID) for reagents and tools used [126,127]. RRIDs are unique and stable, allowing unambiguous identification of reagents or tools used in a study, aiding other researchers to replicate the methods.

Detailed step-by-step procedures can also be saved and shared online, for example, using Protocols.io [128], which assigns a digital object identifier (DOI) to the protocol and allows cross-referencing between protocols and publications.

**Examples**

**Subitem 9a—Example 1**

**Fig 4:**
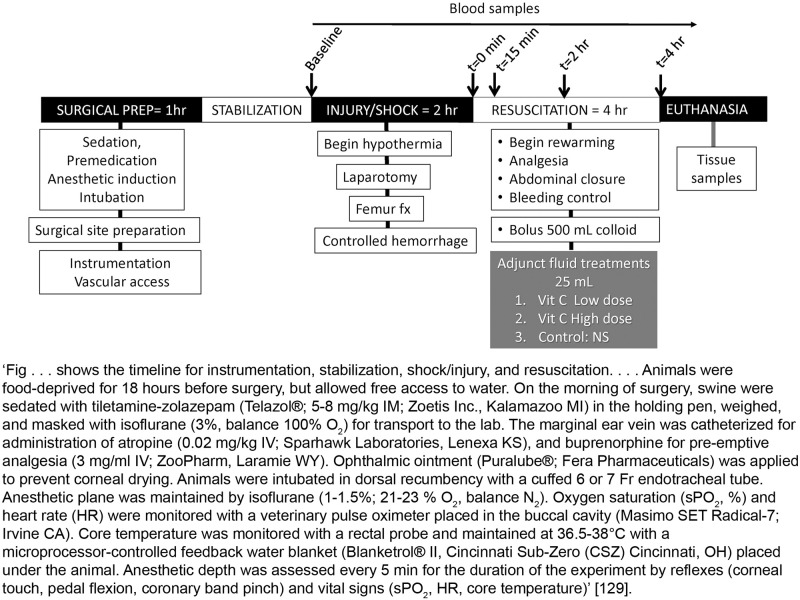
This figure is an alternative version of the figure published in reference [129].

**Subitem 9a—Example 2**

‘For the diet-induced obesity (DIO) model, eight-week-old male mice had *ad libitum* access to drinking water and were kept on standard chow (SFD, 10.9 kJ/g) or on western high-fat diet (HFD; 22 kJ/g; kcal from 42% fat, 43% from carbohydrates and 15% from protein; E15721-34, Ssniff, Soest, Germany) for 15 weeks (https://dx.doi.org/10.17504/protocols.io.kbacsie)’ [130].

**Subitem 9a—Example 3**

‘The frozen kidney tissues were lysed. The protein concentration was determined with the Pierce BCA assay kit (catalogue number 23225; Thermo Fisher Scientific, Rockford, IL, USA). A total of 100–150 μg total proteins were resolved on a 6–12% SDS-PAGE gel. The proteins were then transferred to a nitrocellulose membrane, blocked with 5% skimmed milk for 1 h at room temperature and incubated overnight at 4°C with primary antibodies against the following proteins: proliferating cell nuclear antigen (PCNA; Cat# 2586, RRID: AB_2160343), phospho-AMPK (Cat# 2531, RRID: AB_330330), phospho-mTOR (Cat# 2971, RRID: AB_330970)…. The β-actin (Cat# A5441, RRID: AB_476744) antibody was obtained from Sigma. The blots were subsequently probed with HRP-conjugated anti-mouse (Cat# A0216) or anti-rabbit IgG (Cat# A0208; Beyotime Biotechnology, Beijing, China) at 1:1000. Immunoreactive bands were visualized by enhanced chemiluminescence, and densitometry was performed using ImageJ software (RRID: SCR_003070, Bio-Rad Laboratories)’ [131].

**9b**. **When and how often**.

**Explanation.** Clearly report the frequency and timing of experimental procedures and measurements, including the light and dark cycle (e.g., 12L:12D), circadian time cues (e.g., lights on at 8:00 AM), and experimental time sequence (e.g., interval between baseline and comparator measurements or interval between procedures and measurements). Along with innate circadian rhythms, these can affect research outcomes such as behavioural, physiological, and immunological parameters [132,133]. Also report the timing and frequency of welfare assessments, taking into consideration the normal activity patterns (see Item 16. Animal care and monitoring). For example, nocturnal animals may not show behavioural signs of discomfort during the day [134].

If the timing of procedures or measurements varies between animals, this information can be provided as a supplementary table listing each animal.

**Examples**

**Subitem 9b—Example 1**

‘Blood pressure, heart rate, oxygen saturation and amount of blood extracted were recorded every 5 minutes. Blood samples were drawn at baseline (pre injury), 0 minutes (immediately after injury), and after 30 and 60 minutes’ [135].

**Subitem 9b—Example 2**

‘After a 5-h fast (7:30–12:30am), awake and freely moving mice were randomized and subjected to three consecutive clamps performed in the same mice as described above, with a 2 days recovery after each hyperinsulinemic/hypoglycemic (mHypo, *n* = 6) or hyperinsulinemic/euglycemic (mEugly, *n* = 4) clamps’ [136].

**9c**. **Where (including detail of any acclimatisation periods)**.

**Explanation.** Physiological acclimatisation after a stressful event, such as transport (e.g., between supplier, animal facility, operating theatre, and laboratory), but before the experiment begins allows stabilisation of physiological responses of the animal [137,138]. Protocols vary depending on species, strain, and outcome; for example, physiological acclimatisation following transportation of different animals can take anywhere from 24 hours to more than 1 week [139]. Procedural acclimatisation immediately before a procedure allows stabilisation of the animals’ responses after unaccustomed handling, novel environments, and previous procedures, which otherwise can induce behavioural and physiological changes [140,141]. Standard acclimatisation periods may vary between research laboratories, and this information cannot be inferred by readers.

Indicate where studies were performed (e.g., dedicated laboratory space or animal facility, home cage, open field arena, water maze) and whether periods of physiological or procedural acclimatisation were included in the study protocol, including type and duration. If the study involved multiple sites, explicitly state where each experiment and sample analysis was performed. Include any accreditation of laboratories if appropriate (e.g., if samples were sent to a commercial laboratory for analysis).

**Example**

**Subitem 9c –example 1**

‘Fish were singly housed for 1 week before being habituated to the conditioning tank over 2 consecutive days. The conditioning tank consisted of an opaque tank measuring 20 cm (w) 15 cm (h) 30 cm (l) containing 2.5L of aquarium water with distinct visual cues (spots or stripes) on walls at each end of the tank…. During habituation, each individual fish was placed in the conditioning apparatus for 20 minutes with free access to both compartments and then returned to its home tank’ [142].

**9d**. **Why (provide rationale for procedures)**.

**Explanation.** There may be numerous approaches to investigate any given research problem; therefore, it is important to explain why a particular procedure or technique was chosen. This is especially relevant when procedures are novel or specific to a research laboratory or constrained by the animal model or experimental equipment (e.g., route of administration determined by animal size [143]).

**Examples**

**Subitem 9d—Example 1**

‘Because of the very small caliber of the murine tail veins, partial paravenous injection is common if ^18^F-FDG is administered by tail vein injection (intravenous). This could have significantly biased our comparison of the biodistribution of ^18^F-FDG under various conditions. Therefore, we used intraperitoneal injection of ^18^F-FDG for our experiments evaluating the influence of animal handling on ^18^F-FDG biodistribution’ [144].

**Subitem 9d—Example 2**

‘Since *Xenopus* oocytes have a higher potential for homologous recombination than fertilized embryos… we next tested whether the host transfer method could be used for efficient HDR-mediated knock-in. We targeted the C-terminus of *X*. *laevis* Ctnnb1 (β-catenin), a key cytoskeletal protein and effector of the canonical Wnt pathway, because previous studies have shown that addition of epitope tags to the C-terminus do not affect the function of the resulting fusion protein (Fig …). CRISPR components were injected into *X*. *laevis* oocytes followed by host transfer or into embryos’ [145].

### Item 10. Results

**For each experiment conducted, including independent replications, report**:

**10a**. **Summary/descriptive statistics for each experimental group, with a measure of variability where applicable (e.g., mean and SD, or median and range)**.

**Explanation.** Summary/descriptive statistics provide a quick and simple description of the data; they communicate quantitative results easily and facilitate visual presentation. For continuous data, these descriptors include a measure of central tendency (e.g., mean, median) and a measure of variability (e.g., quartiles, range, standard deviation) to help readers assess the precision of the data collected. Categorical data can be expressed as counts, frequencies, or proportions.

**Fig 5:**
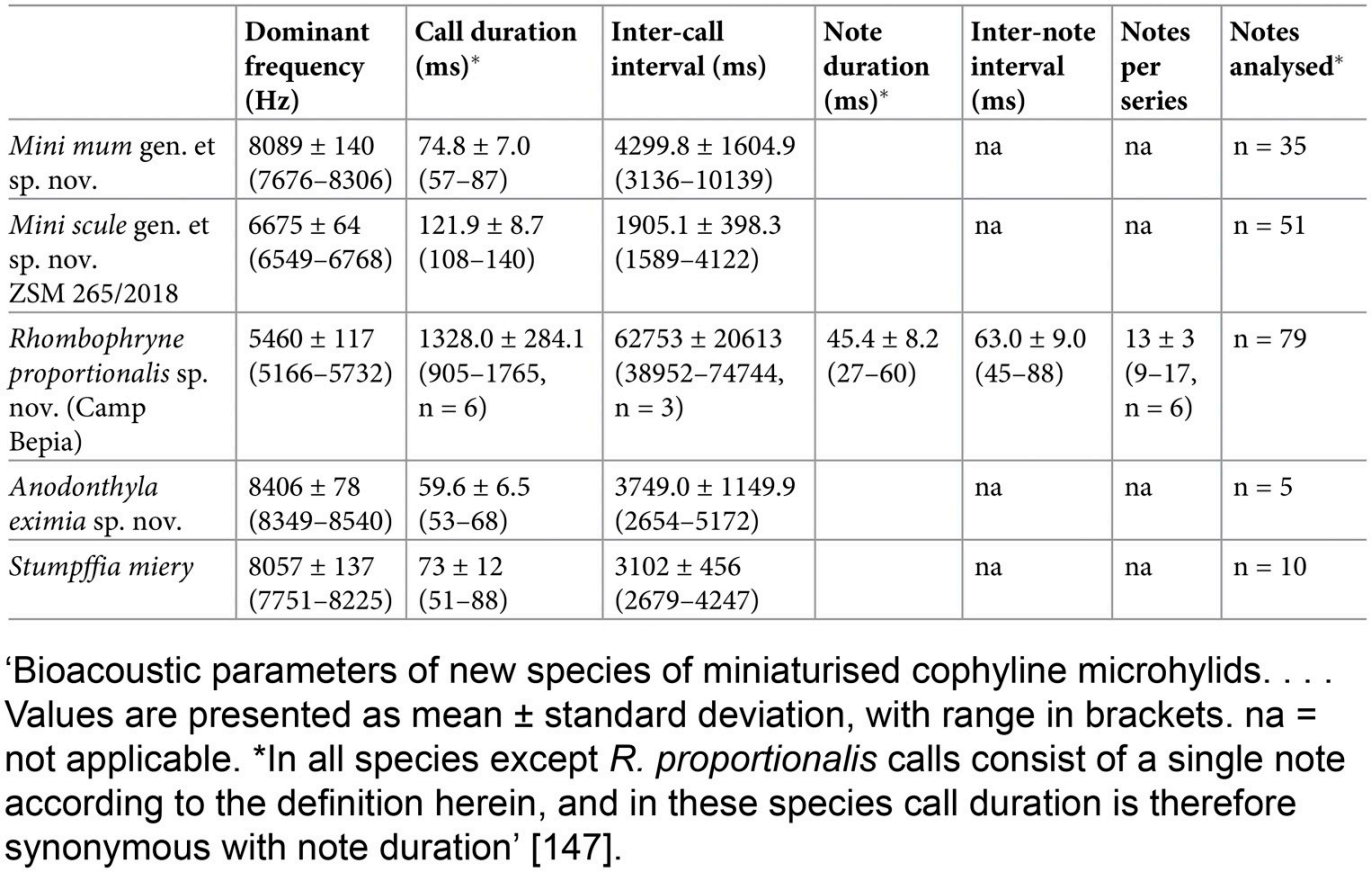
Reproduced from reference [147].

**Fig 6:**
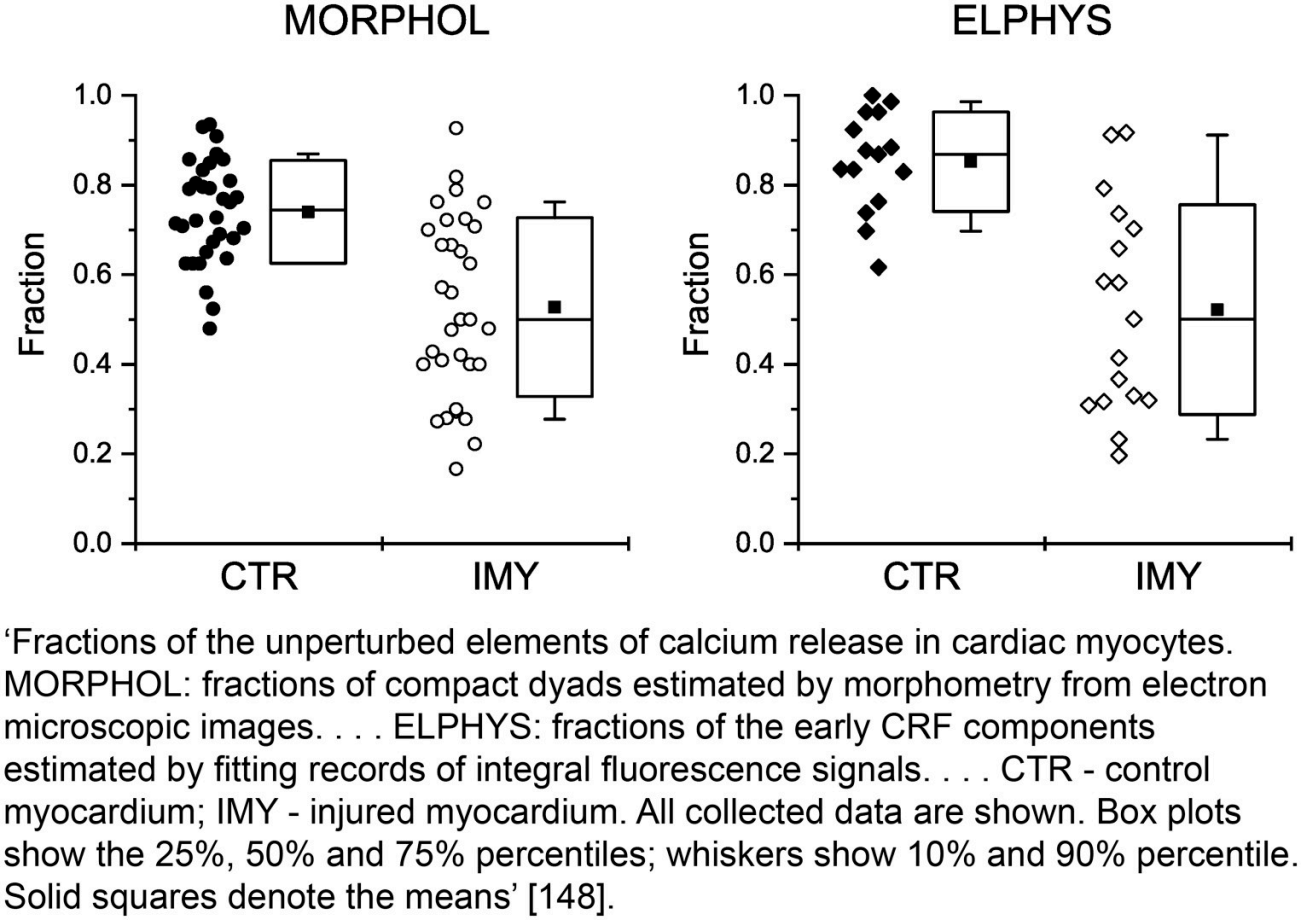
Reproduced from reference [148].

Report data for all experiments conducted. If a complete experiment is repeated on a different day or under different conditions, report the results of all repeats rather than selecting data from representative experiments. Report the exact number of experimental units per group so readers can gauge the reliability of the results (see Item 2. Sample size and Item 3. Inclusion and exclusion criteria). Present data clearly as text, in tables, or in graphs, to enable information to be evaluated or extracted for future meta-analyses [146]. Report descriptive statistics with a clearly identified measure of variability for each group. Fig 5 shows data summarised as means and standard deviations and, in brackets, ranges. Box plots are a convenient way to summarise continuous data, plotted as median and interquartile range, as shown in Fig 6.

**Examples**

**Subitem 10a—Example 1**

**Subitem 10a—Example 2**

**10b**. **If applicable, the effect size with a confidence interval**.

**Explanation.** In hypothesis-testing studies using inferential statistics, investigators frequently confuse statistical significance and small *p*-values with biological or clinical importance [149]. Statistical significance is usually quantified and evaluated against a preassigned threshold, with *p* < 0.05 often used as a convention. However, statistical significance is heavily influenced by sample size and variation in the data (see Item 2. Sample size). Investigators must consider the size of the effect that was observed and whether this is a biologically relevant change.

Effect sizes are often not reported in animal research, but they are relevant to both exploratory and hypothesis-testing studies. An effect size is a quantitative measure that estimates the magnitude of differences between groups or strength of relationships between variables. It can be used to assess the patterns in the data collected and make inferences about the wider population from which the sample came. The confidence interval for the effect indicates how precisely the effect has been estimated and tells the reader about the strength of the effect [150]. In studies in which statistical power is low and/or hypothesis-testing is inappropriate, providing the effect size and confidence interval indicates how small or large an effect might really be, so a reader can judge the biological significance of the data [151,152]. Reporting effect sizes with confidence intervals also facilitates extraction of useful data for systematic review and meta-analysis. When multiple independent studies included in a meta-analysis show quantitatively similar effects, even if each is statistically nonsignificant, this provides powerful evidence that a relationship is ‘real’, although small.

Report all analyses performed, even those providing non-statistically significant results. Report the effect size to indicate the size of the difference between groups in the study, with a confidence interval to indicate the precision of the effect size estimate.

**Example**

**Subitem 10b—Example 1**

**Fig 7:**
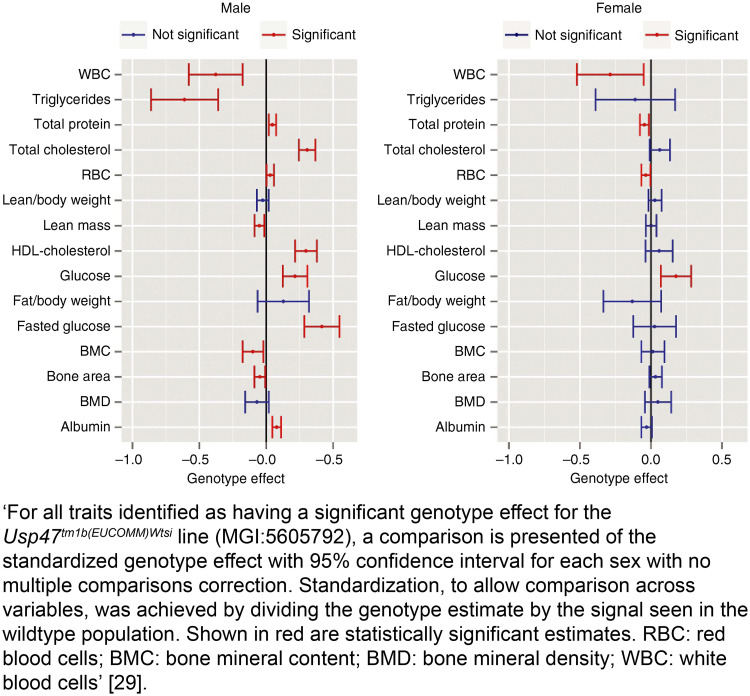
Reproduced from reference [29].

## Recommended Set

The Recommended Set (Box 6) adds context to the study described, including further detail about the methodology and advice on what to include in the more narrative parts of a manuscript. Items are presented in a logical order; there is no ranking within the set.

**Box 6. ARRIVE Recommended Set**

1. Abstract
2. Background
3. Objectives
4. Ethical statement
5. Housing and husbandry
6. Animal care and monitoring
7. Interpretation/scientific implications
8. Generalisability/translation
9. Protocol registration
10. Data access
11. Declaration of interests

### Item 11. Abstract

**Provide an accurate summary of the research objectives, animal species, strain and sex, key methods, principal findings, and study conclusions**.

**Explanation.** A transparent and accurate abstract increases the utility and impact of the manuscript and allows readers to assess the reliability of the study [153]. The abstract is often used as a screening tool by readers to decide whether to read the full article or whether to select an article for inclusion in a systematic review. However, abstracts often either do not contain enough information for this purpose [11] or contain information that is inconsistent with the results in the rest of the manuscript [154,155]. In systematic reviews, initial screens to identify papers are based on titles, abstracts, and keywords [156]. Leaving out of the abstract information such as the species of animal used or the drugs being tested limits the value of preclinical systematic reviews as relevant studies cannot be identified and included. For example, in a systematic review of the effect of the MVA85A vaccine on tuberculosis challenge in animals, the largest preclinical trial did not include the vaccine name in the abstract or keywords of the publication; the paper was only included in the systematic review following discussions with experts in the field [157].

To maximise utility, include details of the species, sex, and strain of animals used and accurately report the methods, results, and conclusions of the study. Also describe the objectives of the study, including whether it was designed either to test a specific hypothesis or to generate a new hypothesis (see Item 13. Objectives). Incorporating this information will enable readers to interpret the strength of evidence and judge how the study fits within the wider knowledge base.

**Examples**

**Item 11—Example 1**

‘Background and Purpose

‘Asthma is an inflammatory disease that involves airway hyperresponsiveness and remodelling. Flavonoids have been associated to anti-inflammatory and antioxidant activities and may represent a potential therapeutic treatment of asthma. Our aim was to evaluate the effects of the sakuranetin treatment in several aspects of experimental asthma model in mice.

‘Experimental Approach

‘Male BALB/c mice received ovalbumin (i.p.) on days 0 and 14, and were challenged with aerolized ovalbumin 1% on days 24, 26 and 28. Ovalbumin-sensitized animals received vehicle (saline and dimethyl sulfoxide, DMSO), sakuranetin (20 mg kg^–1^ per mice) or dexamethasone (5 mg kg^–1^ per mice) daily beginning from 24th to 29th day. Control group received saline inhalation and nasal drop vehicle. On day 29, we determined the airway hyperresponsiveness, inflammation and remodelling as well as specific IgE antibody. RANTES, IL- 5, IL -4, Eotaxin, IL -10, TNF -α, IFN -γ and GMC-SF content in lung homogenate was performed by Bioplex assay, and 8-isoprostane and NF -kB activations were visualized in inflammatory cells by immunohistochemistry.

‘Key Results

‘We have demonstrated that sakuranetin treatment attenuated airway hyperresponsiveness, inflammation and remodelling; and these effects could be attributed to Th2 pro-inflammatory cytokines and oxidative stress reduction as well as control of NF -kB activation.

‘Conclusions and Implications

‘These results highlighted the importance of counteracting oxidative stress by flavonoids in this asthma model and suggest sakuranetin as a potential candidate for studies of treatment of asthma’ [158].

**Item 11—Example 2**

‘In some parts of the world, the laboratory pig (Sus scrofa) is often housed in individual, sterile housing which may impose stress. Our objectives were to determine the effects of isolation and enrichment on pigs housed within the PigTurn^®^—a novel penning system with automated blood sampling—and to investigate tear staining as a novel welfare indicator. Twenty Yorkshire × Landrace weaner pigs were randomly assigned to one of four treatments in a 2 × 2 factorial combination of enrichment (non-enriched [NE] or enriched [E]) and isolation (visually isolated [I] or able to see another pig [NI]). Pigs were catheterised and placed into the PigTurns^®^ 48 h post recovery. Blood was collected automatically twice daily to determine white blood cell (WBC) differential counts and assayed for cortisol. Photographs of the eyes were taken daily and tear staining was quantified using a 0–5 scoring scale and Image-J software to measure stain area and perimeter. Behaviour was video recorded and scan sampled to determine time budgets. Data were analysed as an REML using the MIXED procedure of SAS. Enrichment tended to increase proportion of time standing and lying laterally and decrease plasma cortisol, tear-stain area and perimeter. There was a significant isolation by enrichment interaction. Enrichment given to pigs housed in isolation had no effect on plasma cortisol, but greatly reduced it in non-isolated pigs. Tear-staining area and perimeter were highest in the NE-I treatment compared to the other three treatments. Eosinophil count was highest in the E-NI treatment and lowest in the NE-I treatment. The results suggest that in the absence of enrichment, being able to see another animal but not interact may be frustrating. The combination of no enrichment and isolation maximally impacted tear staining and eosinophil numbers. However, appropriate enrichment coupled with proximity of another pig would appear to improve welfare’ [159].

### Item 12. Background

**12a**. **Include sufficient scientific background to understand the rationale and context for the study, and explain the experimental approach**.

**Explanation.** Scientific background information for an animal study should demonstrate a clear evidence gap and explain why an in vivo approach was warranted. Systematic reviews of the animal literature provide the most convincing evidence that a research question has not been conclusively addressed, by showing the extent of current evidence within a field of research. They can also inform the choice of animal model by providing a comprehensive overview of the models used along with their benefits and limitations [160–162].

Describe the rationale and context of the study and how it relates to other research, including relevant references to previous work. Outline evidence underpinning the hypothesis or objectives and explain why the experimental approach is best suited to answer the research question.

**Example**

**Subitem 12a –example 1**

‘For decades, cardiovascular disease has remained the leading cause of mortality worldwide… [and] cardiovascular research has been performed using healthy and young, non-diseased animal models. Recent failures of cardioprotective therapies in obese insulin-resistant …, diabetic …, metabolic syndrome-affected… and aged… animals that were otherwise successful in healthy animal models has highlighted the need for the development of animal models of disease that are representative of human clinical conditions…. The majority of laboratory-based studies investigating cardiovascular disease and myocardial tolerance to ischemia-reperfusion (I-R) are currently conducted using normogonadic models with either genetically-induced… or diet-induced… obesity and metabolic syndrome (MetS). In the clinical setting, elderly male patients often present with both testosterone deficiency (TD) and MetS…. A strong and compounding association exists between metabolic syndrome and testosterone deficiency which may have significant impact on cardiovascular disease and its outcomes which is not addressed by current models…. Although laboratory investigations generally rely on animal models of isolated metabolic syndrome or hypogonadism, their mutual presentation in the clinical setting warrants the development of appropriate animal models of the MetS with hypogonadism, especially in the context of cardiovascular disease research’ [163].

**12b**. **Explain how the animal species and model used address the scientific objectives and, where appropriate, the relevance to human biology**.

**Explanation.** Provide enough detail for the reader to assess the suitability of the animal model used to address the research question. Include information on the rationale for choosing a particular species and explain how the outcome measures assessed are relevant to the condition under study and how the model was validated. Stating that an animal model is commonly used in the field is not appropriate, and a well-considered, detailed rationale should be provided.

When the study models an aspect of a human disease, indicate how the model is appropriate for addressing the specific objectives of the study [164]. This can include a description of how the induction of the disease, disorder, or injury is sufficiently analogous to the human condition; how the model responds to known clinically effective treatments; how similar symptoms are to the clinical disease; and how animal characteristics were selected to represent the age, sex, and health status of the clinical population [14].

**Examples**

**Subitem 12b—Example 1**

‘For this purpose, we selected a pilocarpine model of epilepsy that is characterized by robust, frequent spontaneous seizures acquired after a brain insult …, well-described behavioral abnormalities …, and poor responses to antiepileptic drugs…. These animals recapitulate several key features of human temporal lobe epilepsy, the most common type of epilepsy in adults’ [165].

**Subitem 12b—Example 2**

‘Transplantation of healthy haematopoietic stem cells (HSCs) is a critical therapy for a wide range of malignant haematological and non-malignant disorders and immune dysfunction…. Zebrafish are already established as a successful model to study the haematopoietic system, with significant homology with mammals…. Imaging of zebrafish transparent embryos remains a powerful tool and has been critical to confirm that the zebrafish Caudal Haematopoietic Tissue (CHT) is comparable to the mammalian foetal haematopoietic niche…. Xenotransplantation in zebrafish embryos has revealed highly conserved mechanisms between zebrafish and mammals. Recently, murine bone marrow cells were successfully transplanted into zebrafish embryos, revealing highly conserved mechanism of haematopoiesis between zebrafish and mammals…. Additionally, CD34 enriched human cells transplanted into zebrafish were shown to home to the CHT and respond to zebrafish stromal-cell derived factors’ [166].

### Item 13. Objectives

**Clearly describe the research question, research objectives and, where appropriate, specific hypotheses being tested**.

**Explanation.** Explaining the purpose of the study by describing the question(s) that the research addresses allows readers to determine whether the study is relevant to them. Readers can also assess the relevance of the model organism, procedures, outcomes measured, and analysis used.

Knowing whether a study is exploratory or hypothesis-testing is critical to its interpretation. A typical exploratory study may measure multiple outcomes and look for patterns in the data or relationships that can be used to generate hypotheses. It may also be a pilot study, which aims to inform the design or feasibility of larger subsequent experiments. Exploratory research helps researchers to design hypothesis-testing experiments by choosing what variables or outcome measures to focus on in subsequent studies.

Testing a specific hypothesis has implications for both the study design and the data analysis [16,167]. For example, an experiment designed to detect a hypothesised effect will likely need to be analysed with inferential statistics, and a statistical estimation of the sample size will need to be performed a priori (see Item 2. Sample size). Hypothesis-testing studies also have a predefined primary outcome measure, which is used to assess the evidence in support of the specific research question (see Item 6. Outcome measures).

In contrast, exploratory research investigates many possible effects and is likely to yield more false positive results because some will be positive by chance. Thus, results from well-designed hypothesis-testing studies provide stronger evidence than those from exploratory or descriptive studies. Independent replication and meta-analysis can further increase the confidence in conclusions.

Clearly outline the objective(s) of the study, including whether it is hypothesis-testing or exploratory, or if it includes research of both types. Hypothesis-testing studies may collect additional information for exploratory purposes; it is important to distinguish which hypotheses were prespecified and which originated after data inspection, especially when reporting unanticipated effects or outcomes that were not part of the original study design.

**Examples**

**Item 13—Example 1**

‘The primary objective of this study was to investigate the cellular immune response to MSC injected into the striatum of allogeneic recipients (6-hydroxydopamine [6-OHDA]-hemilesioned rats, an animal model of Parkinson’s disease [PD]), and the secondary objective was to determine the ability of these cells to prevent nigrostriatal dopamine depletion and associated motor deficits in these animals’ [168].

**Item 13—Example 2**

‘In this exploratory study, we aimed to investigate whether calcium electroporation could initiate an anticancer immune response similar to electrochemotherapy. To this end, we treated immunocompetent balb/c mice with CT26 colon tumors with calcium electroporation, electrochemotherapy, or ultrasound-based delivery of calcium or bleomycin’ [169].

**Item 13—Example 3**

‘While characterizing a *rab-6*.*2*-null *C*. *elegans* strain for another study, we observed that *rab-6*.*2(ok2254)* animals were fragile. We set out to analyze the fragile-skin phenotype in *rab-6*.*2(ok2254)* animals genetically…. We observed several ruptured animals on our *rab-6*.*2(ok2254)* culture plates during normal maintenance, a phenotype very rarely observed in wild-type cultures…. We hypothesized that RAB-6.2 is required for skin integrity’ [170].

### Item 14. Ethical statement

**Provide the name of the ethical review committee or equivalent that has approved the use of animals in this study and any relevant licence or protocol numbers (if applicable)**. **If ethical approval was not sought or granted, provide a justification**.

**Explanation.** Authors are responsible for complying with regulations and guidelines relating to the use of animals for scientific purposes. This includes ensuring that they have the relevant approval for their study from an appropriate ethics committee and/or regulatory body before the work starts. The ethical statement provides editors, reviewers, and readers with assurance that studies have received this ethical oversight [12]. This also promotes transparency and understanding about the use of animals in research and fosters public trust.

Provide a clear statement explaining how the study conforms to appropriate regulations and guidelines. Include the name of the institution where the research was approved and the ethics committee who reviewed it (e.g., Institutional Animal Care and Use Committee [IACUC] in the United States or Animal Welfare and Ethical Review Body [AWERB] in the United Kingdom) and indicate protocol or project licence numbers so that the study can be identified. Also add any relevant accreditation, e.g., American Association for Accreditation of Laboratory Animal Care (AAALAC) [171] or Good Laboratory Practice (GLP).

If the research is not covered by any regulation and formal ethical approval is not required (e.g., a study using animal species not protected by regulations or law), demonstrate that international standards were complied with and cite the appropriate reference. In such cases, provide a clear statement explaining why the research is exempt from regulatory approval.

**Examples**

**Item 14—Example 1**

‘All procedures were conducted in accordance with the United Kingdom Animal (Scientific Procedures) Act 1986, approved by institutional ethical review committees (Alderley Park Animal Welfare and Ethical Review Board and Babraham Institute Animal Welfare and Ethical Review Board) and conducted under the authority of the Project Licence (40/3729 and 70/8307, respectively)’ [172].

**Item 14—Example 2**

‘All protocols in this study were approved by the Committee on the Ethics of Animal Experiments of Fuwai Hospital, Peking Union Medical College and the Beijing Council on Animal Care, Beijing, China (IACUC permit number: FW2010-101523), in compliance with the Guide for the Care and Use of Laboratory Animals published by the US National Institutes of Health (NIH publication no.85-23, revised 1996)’ [173].

**Item 14—Example 3**

‘Samples and data were collected according to Institut de Sélection d’Animale (ISA) protocols, under the supervision of ISA employees. Samples and data were collected as part of routine animal data collection in a commercial breeding program for layer chickens in The Netherlands. Samples and data were collected on a breeding nucleus of ISA for breeding purposes only, and is a non-experimental, agricultural practice, regulated by the Act Animals, and the Royal Decree on Procedures. The Dutch Experiments on Animals Act does not apply to non-experimental, agricultural practices. An ethical review by the Statement Animal Experiment Committee was therefore not required. No extra animal discomfort was caused for sample collection for the purpose of this study’ [174].

### Item 15. Housing and husbandry

**Provide details of housing and husbandry conditions, including any environmental enrichment**.

**Explanation.** The environment determines the health and wellbeing of the animals, and every aspect of it can potentially affect their behavioural and physiological responses, thereby affecting research outcomes [175]. Different studies may be sensitive to different environmental factors, and particular aspects of the environment necessary to report may depend on the type of study [176]. Examples of housing and husbandry conditions known to affect animal welfare and research outcomes are listed in Table 2; consider reporting these elements and any other housing and husbandry conditions likely to influence the study outcomes.

**Table 2:** Examples of information to consider when reporting housing and husbandry, and their effects on laboratory animals.

Information to report	Examples of effects on laboratory animals
Cage/tank/housing system (type and dimensions)	Affects behaviour [177] and fear learning [178]. Tank colour affects stress in aquatic species [179,180].
Food and water (type, composition, supplier, and access)	Affects body weight, tumour development, nephropathy severity [181], and the threshold for developing parkinsonian symptoms [182]. Maternal diet affects offspring body weight [183].
Bedding and nesting material	Affects behavioural responses to stress [184] and pain [185].
Temperature and humidity	Modifies tumour progression [186]. Regulates sexual differentiation in zebrafish [187].
Sanitation (frequency of cage/tank water changes, material transferred, water quality)	Affects blood pressure, heart rate, behaviour [188]. Adds an additional source of variation [189,190].
Social environment (group size and composition/stocking density)	Compromises animal welfare [191]. Induces aggressive behaviour [192,193] and stress [180].
Biosecurity (level)	The microbiological status of animals causes variation in systemic disease parameters [194].
Lighting (type, schedule, and intensity)	Modifies immune and stress responses [195].
Environmental enrichment	Reduces anxiety [196,197], stress [196,197], and abnormal repetitive behaviour [198–200]. Reduces susceptibility to epilepsy [201] and osteoarthritis [202] and modifies the pathology of neurological disorders [203]. Increases foraging behaviour in fish [204].
Sex of the experimenter	Affects physiological stress and pain behaviour [205].

Environment, either deprived or enriched, can affect a wide range of physiological and behavioural responses [206]. Specific details to report include, but are not limited to, structural enrichment (e.g., elevated surfaces, dividers); resources for species-typical activities (e.g., nesting material, shelters, or gnawing sticks for rodents; plants or gravel for aquatic species); and toys or other tools used to stimulate exploration, exercise (e.g., running wheel), and novelty. If no environmental enrichment was provided, this should be clearly stated with justification. Similarly, scientific justification needs to be reported for withholding food and water [207] and for singly housing animals [208,209].

If space is an issue, relevant housing and husbandry details can be provided in the form of a link to the information in a public repository or as supplementary information.

**Examples**

**Item 15—Example 1**

‘Breeding colonies were kept in individually ventilated cages (IVCs; Tecniplast, Italy) at a temperature of 20°C to 24°C, humidity of 50% to 60%, 60 air exchanges per hour in the cages, and a 12/12-hour light/dark cycle with the lights on at 5:30 AM. The maximum caging density was five mice from the same litter and sex starting from weaning. As bedding, spruce wood shavings (Lignocel FS-14; J. Rettenmaier und Soehne GmbH, Rosenberg, Germany) were provided. Mice were fed a standardized mouse diet (1314, Altromin, Germany) and provided drinking water *ad libitum*. All materials, including IVCs, lids, feeders, bottles, bedding, and water were autoclaved before use. Sentinel mice were negative for at least all Federation of laboratory animal science associations (FELASA)-relevant murine infectious agents… as diagnosed by our health monitoring laboratory, mfd Diagnostics GmbH, Wendelsheim, Germany’ [210].

**Item 15—Example 2**

‘Same sex litter mates were housed together in individually ventilated cages with two or four mice per cage. All mice were maintained on a regular diurnal lighting cycle (12:12 light:-dark) with ad libitum access to food (7012 Harlan Teklad LM-485 Mouse/Rat Sterilizable Diet) and water. Chopped corn cob was used as bedding. Environmental enrichment included nesting material (Nestlets, Ancare, Bellmore, NY, USA), PVC pipe, and shelter (Refuge XKA-2450-087, Ketchum Manufacturing Inc., Brockville, Ontario, Canada). Mice were housed under broken barrier-specific pathogen-free conditions in the Transgenic Mouse Core Facility of Cornell University, accredited by AAALAC (The Association for Assessment and Accreditation of Laboratory Animal Care International)’ [211].

### Item 16. Animal care and monitoring

**16a**. **Describe any interventions or steps taken in the experimental protocols to reduce pain, suffering, and distress**.

**Explanation.** A safe and effective analgesic plan is critical to relieve pain, suffering, and distress. Untreated pain can affect the animals’ biology and add variability to the experiment; however, specific pain management procedures can also introduce variability, affecting experimental data [212,213]. Underreporting of welfare management procedures contributes to the perpetuation of noncompliant methodologies and insufficient or inappropriate use of analgesia [213] or other welfare measures. A thorough description of the procedures used to alleviate pain, suffering, and distress provides practical information for researchers to replicate the method.

Clearly describe pain management strategies, including

- specific analgesic
- administration method (e.g., formulation, route, dose, concentration, volume, frequency, timing, and equipment used)
- rationale for the choice (e.g., animal model, disease/pathology, procedure, mechanism of action, pharmacokinetics, personnel safety)
- protocol modifications to reduce pain, suffering, and distress (e.g., changes to the anaesthetic protocol, increased frequency of monitoring, procedural modifications, habituation, etc.)

If analgesics or other welfare measures, reasonably expected for the procedure performed, are not performed for experimental reasons, report the scientific justification [214].

**Examples**

**Subitem 16a—Example 1**

‘If piglets developed diarrhea, they were placed on an electrolyte solution and provided supplemental water, and if the diarrhea did not resolve within 48 h, piglets received a single dose of ceftiofur (5.0 mg ceftiofur equivalent/kg of body weight i.m [Excede, Zoetis, Florham Park, NJ]). If fluid loss continued after treatment, piglets then received a single dose of sulfamethoxazole and trimethoprim oral suspension (50 mg/8 mg per mL, Hi-Tech Pharmacal, Amityville, NY) for 3 consecutive days’ [215].

**Subitem 16a—Example 2**

‘One hour before surgery, we administered analgesia to the mice by offering them nut paste (Nutella; Ferrero, Pino Torinese, Italy) containing 1 mg per kg body weight buprenorphine (Temgesic; Schering-Plough Europe, Brussels, Belgium) for voluntary ingestion, as described previously…. The mice had been habituated to pure nut paste for 2 d prior to surgery’ [216].

**Subitem 16a—Example 3**

‘If a GCPS score equal or greater than 6 (out of 24) was assigned postoperatively, additional analgesia was provided with methadone 0.1 mg kg^−1^ IM (or IV if required) … and pain reassessed 30 minutes later. The number of methadone doses was recorded’ [46].

**16b**. **Report any expected or unexpected adverse events**.

**Explanation.** Reporting adverse events allows other researchers to plan appropriate welfare assessments and minimise the risk of these events occurring in their own studies. If the experiment is testing the efficacy of a treatment, the occurrence of adverse events may alter the balance between treatment benefit and risk [34].

Report any adverse events that had a negative impact on the welfare of the animals in the study (e.g., cardiovascular and respiratory depression, central nervous system disturbance, hypothermia, reduction of food intake). Indicate whether they were expected or unexpected. If adverse events were not observed, or not recorded during the study, explicitly state this.

**Examples**

**Subitem 16b—Example 1**

‘Murine lymph node tumors arose in 11 of 12 mice that received N2-transduced human cells. The *neo* gene could be detected in murine cells as well as in human cells. Significant lymphoproliferation could be seen only in the murine pre-T cells. It took 5 months for murine leukemia to arise; the affected mice displayed symptoms of extreme sickness rapidly, with 5 of the 12 mice becoming moribund on exactly the same day (Figure …), and 6 others becoming moribund within a 1-month period…. Of the 12 mice that had received N2-transduced human cells, 11 had to be killed because they developed visibly enlarged lymph nodes and spleen, hunching, and decrease in body weight, as shown in Figure…. The 12th mouse was observed carefully for 14 months; it did not show any signs of leukemia or other adverse events, and had no abnormal tissues when it was autopsied…. The mice were observed at least once daily for signs of illness, which were defined as any one or more of the following: weight loss, hunching, lethargy, rapid breathing, skin discoloration or irregularities, bloating, hemi-paresis, visibly enlarged lymph nodes, and visible solid tumors under the skin. Any signs of illness were logged as “adverse events” in the experiment, the mouse was immediately killed, and an autopsy was performed to establish the cause of illness’ [217].

**Subitem 16b—Example 2**

‘Although procedures were based on those reported in the literature, dogs under Protocol 1 displayed high levels of stress and many experienced vomiting. This led us to significantly alter procedures in order to optimize the protocol for the purposes of our own fasting and postprandial metabolic studies’ [218].

**16c**. **Describe the humane endpoints established for the study, the signs that were monitored, and the frequency of monitoring**. **If the study did not set humane endpoints, state this**.

**Explanation.** Humane endpoints are predetermined morphological, physiological, and/or behavioural signs that define the circumstances under which an animal will be removed from an experimental study. The use of humane endpoints can help minimise harm while allowing the scientific objectives to be achieved [219]. Report the humane endpoints that were established for the specific study, species, and strain. Include clear criteria of the clinical signs monitored [134] and clinical signs that led to euthanasia or other defined actions. Include details such as general welfare indicators (e.g., weight loss, reduced food intake, abnormal posture) and procedure-specific welfare indicators (e.g., tumour size in cancer studies [50], sensory-motor deficits in stroke studies [220]).

Report the timing and frequency of monitoring, taking into consideration the normal circadian rhythm of the animal and timing of scientific procedures, as well as any increase in the frequency of monitoring (e.g., postsurgery recovery, critical times during disease studies, or following the observation of an adverse event). Publishing score sheets of the clinical signs that were monitored [221] can help guide other researchers to develop clinically relevant welfare assessments, particularly for studies reporting novel procedures.

This information should be reported even if no animal reached any of the humane endpoints. If no humane endpoints were established for the study, explicitly state this.

**Example**

**Subitem 16c—Example 1**

‘Both the research team and the veterinary staff monitored animals twice daily. Health was monitored by weight (twice weekly), food and water intake, and general assessment of animal activity, panting, and fur condition…. The maximum size the tumors allowed to grow in the mice before euthanasia was 2000 mm^3^’ [222].

### Item 17. Interpretation/scientific implications

**17a**. **Interpret the results, taking into account the study objectives and hypotheses, current theory, and other relevant studies in the literature**.

**Explanation.** It is important to interpret the results of the study in the context of the study objectives (see Item 13. Objectives). For hypothesis-testing studies, interpretations should be restricted to the primary outcome (see Item 6. Outcome measures). Exploratory results derived from additional outcomes should not be described as conclusive, as they may be underpowered and less reliable.

Discuss the findings in the context of current theory, ideally with reference to a relevant systematic review, as individual studies do not provide a complete picture. If a systematic review is not available, take care to avoid selectively citing studies that corroborate the results or only those that report statistically significant findings [223].

When appropriate, describe any implications of the experimental methods or research findings for improving welfare standards or reducing the number of animals used in future studies (e.g., the use of a novel approach reduced the results’ variability, thus enabling the use of smaller group sizes without losing statistical power). This may not be the primary focus of the research, but reporting this information enables wider dissemination and uptake of refined techniques within the scientific community.

**Example**

**Subitem 17a—Example 1**

‘This is in contrast to data provided by an ‘intra-renal IL-18 overexpression’ model …, and may reflect an IL-18 concentration exceeding the physiologic range in the latter study’ [224].

**Subitem 17a—Example 2**

‘The new apparatus shows potential for considerably reducing the number of animals used in memory tasks designed to detect potential amnesic properties of new drugs… approximately 43,000 animals have been used in these tasks in the past 5 years but with the application of the continual trials apparatus we estimate that this could have been reduced to 26,000 … with the new paradigm the number of animals needed to obtain reliable results and maintain the statistical power of the tasks is greatly reduced’ [225].

**Subitem 17a—Example 3**

‘In summary, our results show that IL-1Ra protects against brain injury and reduces neuroinflammation when administered peripherally to aged and comorbid animals at reperfusion or 3 hours later. These findings address concerns raised in a recent systematic review on IL-1Ra in stroke… and provide further supporting evidence for IL-1Ra as a lead candidate for the treatment of ischemic stroke’ [226].

**17b**. **Comment on the study limitations, including potential sources of bias, limitations of the animal model, and imprecision associated with the results**.

**Explanation.** Discussing the limitations of the work is important to place the findings in context, interpret the validity of the results, and ascribe a credibility level to its conclusions [227]. Limitations are unavoidable in scientific research, and describing them is essential to share experience, guide best practice, and aid the design of future experiments [228].

Discuss the quality of evidence presented in the study and consider how appropriate the animal model is to the specific research question. A discussion on the rigour of the study design to isolate cause and effect (also known as internal validity [229]) should include whether potential risks of bias have been addressed [9] (see Item 2. Sample size, Item 3. Inclusion and exclusion criteria, Item 4. Randomisation, and Item 5. Blinding).

**Examples**

**Subitem 17b—Example 1**

‘Although in this study we did not sample the source herds, the likelihood of these herds to be IAV positive is high given the commonality of IAV infections in the Midwest…. However, we cannot fully rule out the possibility that new gilts became infected with resident viruses after arrival to the herd. Although new gilts were placed into isolated designated areas and procedures were in place to minimize disease transmission (eg. isolation, vaccination), these areas or procedures might not have been able to fully contain infections within the designated areas’ [230].

**Subitem 17b—Example 2**

‘Even though our data demonstrates that sustained systemic TLR9 stimulation aggravates diastolic HF in our model of gene-targeted diastolic HF, there are several limitations as to mechanistic explanations of causality, as well as extrapolations to clinical inflammatory disease states and other HF conditions. First, our pharmacological inflammatory model does not allow discrimination between effects caused by direct cardiac TLR9 stimulation to that of indirect effects mediated by systemic inflammation. Second, although several systemic inflammatory conditions have disturbances in the innate immune system as important features, and some of these again specifically encompassing distorted TLR9 signalling… sustained TLR9 stimulation does not necessarily represent a clinically relevant inflammatory condition. Finally, the cardiac myocyte SERCA2a KO model does not adequately represent the molecular basis for, or the clinical features of, diastolic HF’ [231].

### Item 18. Generalisability/translation

**Comment on whether, and how, the findings of this study are likely to generalise to other species or experimental conditions, including any relevance to human biology (where appropriate)**.

**Explanation.** An important purpose of publishing research findings is to inform future research. In the context of animal studies, this might take the form of further in vivo research or another research domain (e.g., human clinical trial). Thoughtful consideration is warranted, as additional unnecessary animal studies are wasteful and unethical. Similarly, human clinical trials initiated based on insufficient or misleading animal research evidence increase research waste and negatively influence the risk-benefit balance for research participants [229,232].

Consider the type of study conducted to assess the implications of the findings. Well-designed hypothesis-testing studies provide more robust evidence than exploratory studies (see Item 13. Objectives). Findings from a novel, exploratory study may be used to inform future research in a broadly similar context. Alternatively, enough evidence may have accumulated in the literature to justify further research in another species or in humans. Discuss what (if any) further research may be required to allow generalisation or translation. Discuss and interpret the results in relation to current evidence and, in particular, whether similar [233] or otherwise supportive [234] findings have been reported by other groups. Discuss the range of circumstances in which the effect is observed and factors that may moderate that effect. Such factors could include, for example, the population (e.g., age, sex, strain, species), the intervention (e.g., different drugs of the same class), and the outcome measured (e.g., different approaches to assessing memory).

**Examples**

**Item 18—Example 1**

‘Our results demonstrate that hDBS robustly modulates the mesolimbic network. This finding may hold clinical relevance for hippocampal DBS therapy in epilepsy cases, as connectivity in this network has previously been shown to be suppressed in mTLE. Further research is necessary to investigate potential DBS-induced restoration of MTLE-induced loss of functional connectivity in mesolimbic brain structures’ [235].

**Item 18—Example 2**

‘The tumor suppressor effects of *GAS1* had been previously reported in cell cultures or in xenograft models, this is the first work in which the suppressor activity of murine *Gas1* is reported for primary tumors *in vivo*. Recent advances in the design of safe vectors for transgene delivery… may result in extrapolating our results to humans and so a promising field of research emerges in the area of hepatic, neoplastic diseases’ [236].

### Item 19. Protocol registration

**Provide a statement indicating whether a protocol (including the research question, key design features, and analysis plan) was prepared before the study, and if and where this protocol was registered**.

**Explanation.** Akin to the approach taken for clinical trials, protocol registration has emerged as a mechanism that is likely to improve the transparency of animal research [232,237,238]. Registering a protocol before the start of the experiment enables researchers to demonstrate that the hypothesis, approach, and analysis were planned in advance and not shaped by data as they emerged; it enhances scientific rigour and protects the researcher against concerns about selective reporting of results [239,240]. A protocol should consist of (1) the question being addressed and the key features of the research that is proposed, such as the hypothesis being tested, the primary outcome measure (if applicable), and the statistical analysis plan; and (2) the laboratory procedures to be used to perform the planned experiment.

Protocols may be registered with different levels of completeness. For example, in the Registered Report format offered by an increasing number of journals, protocols undergo peer review, and if accepted, the journal commits to publishing the completed research regardless of the results obtained [237].

Other online resources include the Open Science Framework [241], which is suitable to deposit PHISPS (Population; Hypothesis; Intervention; Statistical Analysis Plan; Primary; Outcome Measure; Sample Size Calculation) protocols [242] and provide researchers with the flexibility to embargo the preregistration, keep it from public view until the research is published, and selectively share it with reviewers and editors. The EDA can also be used to generate a time-stamped PDF, which sets out key elements of the experimental design [19]. This can be used to demonstrate that the study conduct, analysis, and reporting were not unduly driven by emerging data. As a minimum, we recommend registering protocols containing all PHISPS components as outlined above.

Provide a statement indicating whether or not any protocol was prepared before the study, and if applicable, provide the time-stamped protocol or the location of its registration. When there have been deviations from the protocol, describe the rationale for these changes in the publication so that readers can take this into account when assessing the findings.

**Examples**

**Item 19—Example 1**

‘A detailed description of all protocols can be found in the Registered Report (Kandela et al., 2015). Additional detailed experimental notes, data, and analysis are available on the Open Science Framework (OSF) (RRID: SCR_003238) (https://osf.io/xu1g2/)’ [243].

**Item 19—Example 2**

‘To maximize the objectivity of the presented research, we preregistered this study with its 2 hypotheses, its planned methods, and its complete plan of data analysis before the start of data collection (https://osf.io/eb8ua/register/565fb3678c5e4a66b5582f67, accessed 29 December 2017). We closely adhered to our plan…. All statistical analyses closely followed our preregistered analysis plan (https://osf.io/eb8ua/)’ [244].

**Item 19—Example 3**

‘We preregistered our analyses with the Open Science Framework which facilitates reproducibility and open collaboration in science research…. Our preregistration: Sheldon and Griffith (2017), was carried out to limit the number of analyses conducted and to validate our commitment to testing a limited number of a priori hypotheses. Our methods are consistent with this preregistration …’ [245].

### Item 20. Data access

**Provide a statement describing if and where study data are available**.

**Explanation.** A data-sharing statement describes how others can access the data on which the paper is based. Sharing adequately annotated data allows others to replicate data analyses so that results can be independently tested and verified. Data sharing allows the data to be repurposed and new datasets to be created by combining data from multiple studies (e.g., to be used in secondary analyses). This allows others to explore new topics and increases the impact of the study, potentially preventing unnecessary use of animals and providing more value for money. Access to raw data also facilitates text and automated data mining [246].

An increasing number of publishers and funding bodies require authors or grant holders to make their data publicly available [247]. Journal articles with accompanying data may be cited more frequently [248,249]. Datasets can also be independently cited in their own right, which provides additional credit for authors. This practice is gaining increasing recognition and acceptance [250].

When possible, make available all data that contribute to summary estimates or claims presented in the paper. Data should follow the FAIR guiding principles [251]; that is, data are findable, accessible (i.e., do not use outdated file types), interoperable (can be used on multiple platforms and with multiple software packages), and reusable (i.e., have adequate data descriptors).

Data can be made publicly available via a structured, specialised (domain-specific), open-access repository such as those maintained by the National Center for Biotechnology Information (NCBI, https://www.ncbi.nlm.nih.gov/) or European Bioinformatics Institute (EBI, https://www.ebi.ac.uk/). If such a repository is not available, data can be deposited in unstructured but publicly available repositories (e.g., Figshare [https://figshare.com/], Dryad [https://datadryad.org/], Zenodo [https://zenodo.org/], or Open Science Framework [https://osf.io/]). There are also search platforms to identify relevant repositories with rigorous standards, e.g., FairSharing (https://fairsharing.org/) and re3data (https://www.re3data.org/).

**Examples**

**Item 20—Example 1**

‘Data Availability: All data are available from Figshare at http://dx.doi.org/10.6084/m9.figshare.1288935’ [252].

**Item 20—Example 2**

‘A fundamental goal in generating this dataset is to facilitate access to spiny mouse transcript sequence information for external collaborators and researchers. The sequence reads and metadata are available from the NCBI (PRJNA342864) and assembled transcriptomes (Trinity_v2.3.2 and tr2aacds_v2) are available from the Zenodo repository (https://doi.org/10.5281/zenodo.808870), however accessing and utilizing this data can be challenging for researchers lacking bioinformatics expertise. To address this problem we are hosting a SequenceServer… BLAST-search website (http://spinymouse.erc.monash.edu/sequenceserver/). This resource provides a user-friendly interface to access sequence information from the tr2aacds_v2 assembly (to explore annotated protein-coding transcripts) and/or the Trinity_v2.3.2 assembly (to explore non-coding transcripts)’ [253].

### Item 21. Declaration of interests

**21a**. **Declare any potential conflicts of interest, including financial and nonfinancial**. **If none exist, this should be stated**.

**Explanation.** A competing or conflict of interest is anything that interferes with (or could be perceived as interfering with) the full and objective presentation, analysis, and interpretation of the research. Competing or conflicts of interest can be financial or nonfinancial, professional or personal. They can exist in institutions, in teams, or with individuals. Potential competing interests are considered in peer review, editorial, and publication decisions; the aim is to ensure transparency, and in most cases, a declaration of a conflict of interest does not obstruct the publication or review process.

Examples are provided in Box 7. If unsure, declare all potential conflicts, including both perceived and real conflicts of interest [254].

**Box 7. Examples of competing or conflicts of interest**

**Financial**

Funding and other payments received or expected by the authors directly arising from the publication of the study, or funding or other payments from an organisation with an interest in the outcome of the work.

**Nonfinancial**

Research that may benefit the individual or institution in terms of goods in kind. This includes unpaid advisory position in a government, nongovernment organisation, or commercial organisations.

**Affiliations**

Employed by, on the advisory board, or a member of an organisation with an interest in the outcome of the work.

**Intellectual property**

Patents or trademarks owned by someone or their organisation. This also includes the potential exploitation of the scientific advance being reported for the institution, the authors, or the research funders.

**Personal**

Friends, family, relationships, and other close personal connections to people who may potentially benefit financially or in other ways from the research.

**Ideology**

Beliefs or activism (e.g., political or religious) relevant to the work. Membership of a relevant advocacy or lobbying organisation.

**Examples**

**Subitem 21a—Example 1**

‘The study was funded by Gubra ApS. LSD, PJP, GH, KF and HBH are employed by Gubra ApS. JJ and NV are the owners of Gubra ApS. Gubra ApS provided support in the form of materials and salaries for authors LSD, PJP, GH, KF, HBH, JJ and NV’ [255].

**Subitem 21a—Example 2**

‘The authors have declared that no competing interests exist’ [256].

**21b**. **List all funding sources (including grant identifier) and the role of the funder(s) in the design, analysis, and reporting of the study**.

**Explanation.** The identification of funding sources allows the reader to assess any competing interests and any potential sources of bias. For example, bias, as indicated by a prevalence of more favourable outcomes, has been demonstrated for clinical research funded by industry compared with studies funded by other sources [257–259]. Evidence for preclinical research also indicates that funding sources may influence the interpretation of study outcomes [254,260].

Report the funding information including the financial supporting body(s) and any grant identifier(s). If the study was supported by several sources of funding, list them all, including internal grants. Specify the role of the funder in the design, analysis, reporting, and/or decision to publish. If the research did not receive specific funding but was performed as part of the employment of the authors, name the employer.

**Examples**

**Subitem 21b—Example 1**

‘Support was provided by the Italian Ministry of Health: Current research funds PRC 2010/001 [http://www.salute.gov.it/] to MG. The funders had no role in study design, data collection and analysis, decision to publish, or preparation of the manuscript’ [261].

**Subitem 21b—Example 2**

‘This study was financially supported by the Tuberculosis and Lung Research Center of Tabriz University of Medical Sciences and the Research Council of University of Tabriz. The funders had no role in study design, data collection and analysis, decision to publish, or preparation of the manuscript’ [262].

**Subitem 21b—Example 3**

‘This work was supported by the salary paid to AEW. The funder had no role in study design, data collection and analysis, decision to publish, or preparation of the manuscript’ [263].

## Supporting information

S1 Annotated Byline Individual authors’ positions at the time this article was submitted. (DOCX)

S1 Annotated References Further context on the works cited in this article. (DOCX)

## Abbreviations

AAALAC: American Association for Accreditation of Laboratory Animal Care
ARRIVE: Animal Research: Reporting of In Vivo Experiments
AVMA: American Veterinary Medical Association
AWERB: Animal Welfare and Ethical Review Body
DOI: digital object identifier
EBI: European Bioinformatics Institute
EDA: Experimental Design Assistant
GLP: Good Laboratory Practice
IACUC: Institutional Animal Care and Use Committee
NC3Rs: National Centre for the 3Rs
NCBI: National Center for Biotechnology Information
PHISPS: Population; Hypothesis; Intervention; Statistical Analysis Plan; Primary; Outcome Measure; Sample Size Calculation
RRID: Research Resource Identifier
SAMPL: Statistical Analyses and Methods in the Published Literature
SPF: Specific Pathogen Free
