# Supplementary material for: Reporting animal research: Explanation and elaboration for the ARRIVE guidelines 2.0
Source: PLoS Biol. 2020 Jul 14;18(7):e3000411. doi: 10.1371/journal.pbio.3000411 (PMC7360025; doi:10.1371/journal.pbio.3000411)
Supplement: S1 Annotated Byline — (DOCX) [file pbio.3000411.s001.docx]

Nathalie Percie du Sert^1^*, Amrita Ahluwalia^2,3^, Sabina Alam^4^, Marc T. Avey^5^, Monya Baker^6^, William J. Browne^7^, Alejandra Clark^8^, Innes C. Cuthill^9^, Ulrich Dirnagl^10^, Michael Emerson^11^, Paul Garner^12^, Stephen T. Holgate^13^, David W. Howells^14^, Viki Hurst^15^, Natasha A. Karp^16^, Stanley E. Lazic^17^, Katie Lidster^18^, Catriona J. MacCallum^19^, Malcolm Macleod^20,21^, Esther J. Pearl^22^, Ole H. Petersen^23^, Frances Rawle^24^, Penny Reynolds^25^, Kieron Rooney^26^, Emily S. Sena^27^, Shai D. Silberberg^28^, Thomas Steckler^29^, Hanno Würbel^30^

1 Head of Experimental Design and Reporting, NC3Rs, London, United Kingdom,

2 Professor of Vascular Pharmacology, Co-Director, The William Harvey Research Institute, London, United Kingdom,

3 Director of the Barts Cardiovascular CTU, Queen Mary University of London, London, United Kingdom,

4 Director of Publishing Ethics and Integrity, Taylor & Francis Group, London, United Kingdom,

5 Lead Health Scientist, Health Science Practice, ICF, Durham, North Carolina, United States of America,

6 Senior Editor, Opinion, Nature, San Francisco, California, United States of America,

7 Professor of Statistics, School of Education, University of Bristol, Bristol, United Kingdom,

8 Senior Editor, Team Manager – Life Sciences, PLOS ONE, Cambridge, United Kingdom,

9 Professor of Behavioural Ecology, School of Biological Sciences, University of Bristol, Bristol, United Kingdom,

10 Director, QUEST Center for Transforming Biomedical Research, Berlin Institute of Health & Department of Experimental Neurology, Charite Universitätsmedizin Berlin, Berlin, Germany,

11 Reader in Platelet Pharmacology, National Heart and Lung Institute, Imperial College London, London, United Kingdom,

12 Professor and Director of the Centre for Evidence Synthesis in Global Health, Clinical Sciences Department, Liverpool School of Tropical Medicine, Liverpool, United Kingdom,

13 MRC Clinical Professor, Clinical and Experimental Sciences, University of Southampton, Southampton, United Kingdom,

14 Professor of Neuroscience and Brain Plasticity, Tasmanian School of Medicine, University of Tasmania, Hobart, Australia,

15 Science Manager – Experimental Design and Reporting, NC3Rs, London, United Kingdom,

16 Principal Scientist – Statistician & UK Team Lead, Data Sciences & Quantitative Biology, Discovery Sciences, R&D, AstraZeneca, Cambridge, United Kingdom,

17 Chief Scientific Officer, Prioris.ai Inc, Ottawa, Canada,

18 Programme Manager – Animal Welfare, NC3Rs, London, United Kingdom,

19 Director of Open Science, Hindawi Ltd, London, United Kingdom,

20 Professor of Neurology and Translational Neuroscience, Centre for Clinical Brain Sciences, University of Edinburgh, Edinburgh, United Kingdom,

21 Academic Lead for Research Improvement and Research Integrity, University of Edinburgh, Edinburgh, United Kingdom,

22 Programme Manager – Experimental Design, NC3Rs, London, United Kingdom,

23 Director of the Academia Europaea Knowledge Hub, Cardiff University, Cardiff, United Kingdom,

24 Director of Policy, Ethics and Governance, Medical Research Council, London, United Kingdom,

25 Biostatistician, Statistics in Anesthesiology Research (STAR) Core & Research Assistant Professor, Department of Anesthesiology College of Medicine, University of Florida, Gainesville, Florida, United States of America,

26 Associate Professor, Discipline of Exercise and Sport Science, Faculty of Medicine and Health, University of Sydney, Sydney, Australia,

27 Stroke Association Kirby Laing Foundation Senior Non-Clinical Lecturer, Centre for Clinical Brain Sciences, University of Edinburgh, Edinburgh, United Kingdom,

28 Director of Research Quality, National Institute of Neurological Disorders and Stroke, Bethesda, Maryland, United States of America,

29 Associate Director, BRQC Animal Welfare Strategy Lead, Janssen Pharmaceutica NV, Beerse, Belgium,

30 Professor for Animal Welfare, Veterinary Public Health Institute, Vetsuisse Faculty, University of Bern, Bern, Switzerland
