# Supplementary material for: Reporting animal research: Explanation and elaboration for the ARRIVE guidelines 2.0
Source: PLoS Biol. 2020 Jul 14;18(7):e3000411. doi: 10.1371/journal.pbio.3000411 (PMC7360025; doi:10.1371/journal.pbio.3000411)
Supplement: S1 Annotated References — (DOCX) [file pbio.3000411.s002.docx]

## **Annotated references**

14. van der Worp HB, Howells DW, Sena ES, Porritt MJ, Rewell S, O'Collins V, et al. Can animal models of disease reliably inform human studies? PLoS Med. 2010;7(3):e1000245. doi: 10.1371/journal.pmed.1000245.

***** Further information on potential sources of bias in experiments, including exclusion of animals and data points**

16. Festing MF, Altman DG. Guidelines for the design and statistical analysis of experiments using laboratory animals. ILAR J. 2002;43(4):244-58. Epub 2002/10/23. PubMed PMID: 12391400.

***** Further information on experimental design and statistical analysis for animal studies**

17. Bate ST, Clark RA. The design and statistical analysis of animal experiments. Cambridge, United Kingdom: Cambridge University Press; 2014.

***** Comprehensive resource on experimental design and statistical analysis for animal studies**

24. Lazic SE, Clarke-Williams CJ, Munafò MR. What exactly is ‘N’ in cell culture and animal experiments? PLoS Biol. 2018;16(4):e2005282. doi: 10.1371/journal.pbio.2005282.

***** Further information on identifying the experimental unit**

39. Peng C-YJ, Long H, Abaci S. Power analysis software for educational researchers. The Journal of Experimental Education. 2012;80(2):113-36. doi: 10.1080/00220973.2011.647115.

***** A review of software that can be used for sample size calculation**

45. Bate ST. How to decide your sample size when the power calculation is not straightforward. 2018 Aug 1 [cited 2018 Aug 2]. In: NC3Rs.org.uk [Internet]. Available from: https://www.nc3rs.org.uk/news/how-decide-your-sample-size-when-power-calculation-not-straightforward.

***** Advice on deciding sample size when a power calculation is not straightforward**

54. Kang H. The prevention and handling of the missing data. Korean J Anesthesiol. 2013;64(5):402-6. Epub 2013/06/07. doi: 10.4097/kjae.2013.64.5.402. PubMed PMID: 23741561; PubMed Central PMCID: PMCPMC3668100.

***** Further information on techniques to handle missing data**

67. Hirst JA, Howick J, Aronson JK, Roberts N, Perera R, Koshiaris C, et al. The need for randomization in animal trials: an overview of systematic reviews. PLoS ONE. 2014;9(6):e98856. doi: 10.1371/journal.pone.0098856.

***** An overview of systematic reviews showing importance of randomisation and blinding to reduce bias, especially for subjective outcomes**

68. Vesterinen HM, Sena ES, ffrench-Constant C, Williams A, Chandran S, Macleod MR. Improving the translational hit of experimental treatments in multiple sclerosis. Multiple Sclerosis Journal. 2010;16(9):1044-55. doi: doi:10.1177/1352458510379612. PubMed PMID: 20685763.

***** A systematic review of animal models of multiple sclerosis, demonstrating the importance of randomisation and blinding to prevent overestimation of treatment effect**

73. Kang M, Ragan BG, Park JH. Issues in outcomes research: an overview of randomization techniques for clinical trials. J Athl Train. 2008;43(2):215-21. Epub 2008/03/18. doi: 10.4085/1062-6050-43.2.215. PubMed PMID: 18345348; PubMed Central PMCID: PMCPMC2267325.

***** Further reading on randomisation strategies, including block and stratified randomisation**

74. Altman DG, Bland JM. Treatment allocation by minimisation. BMJ. 2005;330(7495):843. Epub 2005/04/09. doi: 10.1136/bmj.330.7495.843. PubMed PMID: 15817555; PubMed Central PMCID: PMCPMC556084.

***** Further reading on randomisation strategies, including minimisation**

93. Landis SC, Amara SG, Asadullah K, Austin CP, Blumenstein R, Bradley EW, et al. A call for transparent reporting to optimize the predictive value of preclinical research. Nature. 2012;490(7419):187-91. Epub 2012/10/13. doi: 10.1038/nature11556. PubMed PMID: 23060188; PubMed Central PMCID: PMCPMC3511845.

***** NIH standards for reporting preclinical research**

95. Munafò MR, Nosek BA, Bishop DVM, Button KS, Chambers CD, Percie du Sert N, et al. A manifesto for reproducible science. Nature Human Behaviour. 2017;1:0021. doi: 10.1038/s41562-016-0021.

***** Futher information on improving research quality through improving methods, reporting and dissemination, transparency and open science, peer review and incentives**

98. Lang TA, Altman DG. Basic statistical reporting for articles published in biomedical journals: the "Statistical Analyses and Methods in the Published Literature" or the SAMPL Guidelines. Int J Nurs Stud. 2015;52(1):5-9. Epub 2014/12/03. doi: 10.1016/j.ijnurstu.2014.09.006. PubMed PMID: 25441757.

***** Further guidance on reporting statistical analysis methods**

108. Jackson SJ, Andrews N, Ball D, Bellantuono I, Gray J, Hachoumi L, et al. Does age matter? The impact of rodent age on study outcomes. Lab Anim. 2017;51(2):160-9. doi: 10.1177/0023677216653984. PubMed PMID: 27307423.

***** Further information on the importance of selecting and reporting animal age in rodent studies**

113. Baker DG. Natural pathogens of laboratory mice, rats, and rabbits and their effects on research. Clin Microbiol Rev. 1998;11(2):231-66. doi: 10.1128/cmr.11.2.231.

***** Further information on the effects of pathogens on both animal health and research**

116. Mallapaty S. In the name of reproducibility. Lab Animal. 2018;47(7):178-81. doi: 10.1038/s41684-018-0095-7.

***** Further information on the importance of accurately identifying strains and lines using correct nomenclature**

127. Bandrowski A, Brush M, Grethe JS, Haendel MA, Kennedy DN, Hill S, et al. The Resource Identification Initiative: a cultural shift in publishing. J Comp Neurol. 2016;524(1):8-22. Epub 2015/11/26. doi: 10.1002/cne.23913. PubMed PMID: 26599696; PubMed Central PMCID: PMCPMC4684178.

***** Futher information on using RRIDs to unambigously identify resources used**

134. Hawkins P, Morton DB, Burman O, Dennison N, Honess P, Jennings M, et al. A guide to defining and implementing protocols for the welfare assessment of laboratory animals: eleventh report of the BVAAWF/FRAME/RSPCA/UFAW Joint Working Group on Refinement. Lab Anim. 2011;45(1):1-13. doi: 10.1258/la.2010.010031.

***** Practical guidance for assessing and monitoring laboratory animal welfare**

139. Obernier JA, Baldwin RL. Establishing an appropriate period of acclimatization following transportation of laboratory animals. ILAR Journal. 2006;47(4):364-9. doi: 10.1093/ilar.47.4.364.

***** Further reading on the importance of acclimatisation for research animals following transport**

152. Nakagawa S, Cuthill IC. Effect size, confidence interval and statistical significance: a practical guide for biologists. Biol Rev Camb Philos Soc. 2007;82(4):591-605. Epub 2007/10/20. doi: 10.1111/j.1469-185X.2007.00027.x. PubMed PMID: 17944619.

***** Further information on confidence intervals, including programs and websites that can calculate them**

162. Sena ES, Currie GL, McCann SK, Macleod MR, Howells DW. Systematic reviews and meta-analysis of preclinical studies: why perform them and how to appraise them critically. J Cereb Blood Flow Metab. 2014;34(5):737-42. Epub 2014/02/20. doi: 10.1038/jcbfm.2014.28. PubMed PMID: 24549183; PubMed Central PMCID: PMCPMC4013765.

***** Further information on systematic reviews of animal research**

167. Kimmelman J, Mogil JS, Dirnagl U. Distinguishing between exploratory and confirmatory preclinical research will improve translation. PLoS Biol. 2014;12(5):e1001863. doi: 10.1371/journal.pbio.1001863.

***** Further information on differentiating exploratory from hypothesis-testing research, including the implications of each research type for both experimental design and drawing conclusions from the resulting research**

176. Guidance for the description of animal research in scientific publications. ILAR J. 2014;55(3):536-40. doi: 10.1093/ilar/ilu070.

***** Further information on aspects of housing and husbandry that can affect experimental results**

212. Jirkof P. Side effects of pain and analgesia in animal experimentation. Lab Anim (NY). 2017;46(4):123-8. Epub 2017/03/23. doi: 10.1038/laban.1216. PubMed PMID: 28328895.

***** Further information on the effects of both untreated pain and analgesics in animal experiments**

221. Morton DB. A systematic approach for establishing humane endpoints. ILAR J. 2000;41(2):80-6. doi: 10.1093/ilar.41.2.80.

***** Further information on establishing humane endpoints and using observation score sheets**

227. Ioannidis JP. Limitations are not properly acknowledged in the scientific literature. J Clin Epidemiol. 2007;60(4):324-9. Epub 2007/03/10. doi: 10.1016/j.jclinepi.2006.09.011. PubMed PMID: 17346604.

***** Further information on reporting limitations**

233. Voelkl B, Vogt L, Sena ES, Würbel H. Reproducibility of preclinical animal research improves with heterogeneity of study samples. PLoS Biol. 2018;16(2):e2003693. doi: 10.1371/journal.pbio.2003693.

***** Further information on the importance for generalisability of evidence from heterogeneous populations**

234. Munafò MR, Davey Smith G. Robust research needs many lines of evidence. Nature. 2018;553(7689):399-401. Epub 2018/01/26. doi: 10.1038/d41586-018-01023-3. PubMed PMID: 29368721.

***** Further information on the importance of different evidence supporting the same conclusions**

237. Chambers CD, Feredoes E, Muthukumaraswamy SD, Etchells PJ. Instead of "playing the game" it is time to change the rules: Registered Reports at AIMS Neuroscience and beyond. AIMS Neuroscience. 2014;1(1):4-17. doi: DOI: 10.3934/Neuroscience2014.1.4.

***** Further information on the Registered Reports format**

241. Nosek BA, Ebersole CR, DeHaven AC, Mellor DT. The preregistration revolution. Proc Natl Acad Sci U S A. 2018;115(11):2600-6. Epub 2018/03/14. doi: 10.1073/pnas.1708274114. PubMed PMID: 29531091; PubMed Central PMCID: PMCPMC5856500.

***** Further information about protocol registration including practical ways to implement preregistration in different research scenarios**

251. Wilkinson MD, Dumontier M, Aalbersberg IJ, Appleton G, Axton M, Baak A, et al. The FAIR Guiding Principles for scientific data management and stewardship. Scientific Data. 2016;3:160018. doi: 10.1038/sdata.2016.18.

***** Further information on FAIR data (findable, accessible, interoperable and reusable)**

254. Bero L, Anglemyer A, Vesterinen H, Krauth D. The relationship between study sponsorship, risks of bias, and research outcomes in atrazine exposure studies conducted in non-human animals: Systematic review and meta-analysis. Environ Int. 2016;92-93:597-604. doi: 10.1016/j.envint.2015.10.011.

***** An animal research example highlighting importance of reporting research sponsorship**

257. Lundh A, Sismondo S, Lexchin J, Busuioc OA, Bero L. Industry sponsorship and research outcome. Cochrane Database Syst Rev. 2012;(12). doi: 10.1002/14651858.MR000033.pub2. PubMed PMID: MR000033.

***** A clinical research example highlighting importance of reporting research sponsorship**
